# Genome-wide analysis reveals adaptation to high altitudes in Tibetan sheep

**DOI:** 10.1038/srep26770

**Published:** 2016-05-27

**Authors:** Caihong Wei, Huihua Wang, Gang Liu, Fuping Zhao, James W. Kijas, Youji Ma, Jian Lu, Li Zhang, Jiaxue Cao, Mingming Wu, Guangkai Wang, Ruizao Liu, Zhen Liu, Shuzhen Zhang, Chousheng Liu, Lixin Du

**Affiliations:** 1 Institute of Animal Sciences, Chinese Academy of Agricultural Sciences, National Center for Molecular Genetics and Breeding of Animal, Beijing, People’s Republic of China; 2 National Animal Husbandry Service, National Center of Preservation & Utilization of Animal Genetic Resources, Beijing, People’s Republic of China; 3 Institute of apicultural research, Chinese Academy of Agricultural Sciences, Beijing, People’s Republic of China; 4 Livestock Industries, CSIRO, Brisbane, Australia; 5 College of Animal Science and Technology, Gansu Agriculture University, Lanzhou 730070, People’s Republic of China

## Abstract

Tibetan sheep have lived on the Tibetan Plateau for thousands of years; however, the process and consequences of adaptation to this extreme environment have not been elucidated for important livestock such as sheep. Here, seven sheep breeds, representing both highland and lowland breeds from different areas of China, were genotyped for a genome-wide collection of single-nucleotide polymorphisms (SNPs). The *F*_*ST*_ and XP-EHH approaches were used to identify regions harbouring local positive selection between these highland and lowland breeds, and 236 genes were identified. We detected selection events spanning genes involved in angiogenesis, energy production and erythropoiesis. In particular, several candidate genes were associated with high-altitude hypoxia, including *EPAS1, CRYAA, LONP1, NF1, DPP4, SOD1, PPARG* and *SOCS2. EPAS1* plays a crucial role in hypoxia adaption; therefore, we investigated the exon sequences of *EPAS1* and identified 12 mutations. Analysis of the relationship between blood-related phenotypes and *EPAS1* genotypes in additional highland sheep revealed that a homozygous mutation at a relatively conserved site in the *EPAS1* 3′ untranslated region was associated with increased mean corpuscular haemoglobin concentration and mean corpuscular volume. Taken together, our results provide evidence of the genetic diversity of highland sheep and indicate potential high-altitude hypoxia adaptation mechanisms, including the role of *EPAS1* in adaptation.

The Tibetan Plateau represents 25% of the landmass of China. It is the largest high-altitude area on earth, with an average altitude exceeding 4,500 m^1^. Such a high-altitude environment has produced many unique highland species. Compared with neighbouring and lowland populations, the native people and animals have adapted to this habitat, which has a 43% lower partial pressure of oxygen^2^ and 40% higher ultraviolet radiation^3^. Several animals, such as Tibetan antelopes^4^ and Tibetan Mastiffs^5^,^6^, have recently been shown to exhibit adaptation to living in this challenging environment. China has many sheep breeds, such as Tibetan and Mongolian sheep, that are widely distributed from the highlands to lowlands, many of which have evolved over centuries or even millennia^7^,^8^. Therefore, they also represent ideal organisms to study plateau adaptability.

To date, the genome sequences of Tibetan antelopes^4^, Tibetan wild boars^9^, yaks^10^ and ground tits^11^ have been generated, and several important pathways and functional categories have been identified, including energy metabolism and oxygen transmission, response to hypoxia, DNA repair and ATPase production. In addition, the mechanisms underlying plateau adaptability have been explored using population surveys of SNP data, successfully identifying candidate genes for genetic adaptation to the Tibetan Plateau. Recent studies have demonstrated that the positively selected haplotypes of *EGLN1* and *PPARA* were significantly associated with the low haemoglobin content of Tibetan people, which is a unique characteristic of this extreme high-altitude population^12^. One single-nucleotide polymorphism (SNP) in *EPAS1*, encoding a transcription factor involved in response to hypoxia, was identified; for this SNP, there was a 78% frequency difference between the Tibetan and Han populations, representing the largest allele frequency difference observed for any human gene to date^13^. Recently, variations in *EPAS1* were identified as high-altitude adaptations in the Tibetan Mastiff 5,6. Additionally, other hypoxia-related genes were identified in yaks (*ADAM17, ARG2* and *MMP3*), Tibetan antelopes (*ADORA2A, CCL2, ENG, PIK3C2A, PKLR, ATP12A* and *NOS3*) and Tibetan wild boars (*ALB, ECE1, GNG2* and *PIK3C2G*)^4^,^9^,^10^. In addition, Qu found 11 candidate positively selected genes (*HIFAN, MTOR, SRF, TXNRD2, WNT7B, ANGP4, ADAM9, PSMD2, LRRC7, MDH1B* and *LRRK1*) associated with a hypoxia response in ground tits^11^. Almost all of the identified genes mentioned above belong to the list of 247 hypoxia genes that are priority candidates for adaptation to high-altitude hypoxia^12^; however, each species may have different candidate genes. This phenomenon might indicate differences between species in the specific genes selected, even in the same environment. Thus, Tibetan sheep might differ from other species with respect to the molecular mechanisms of high-altitude adaptation. However, there has been no research on the adaptation of Tibetan sheep to the high-altitude plateau.

Data from several amphibians and mammals have indicated that modifications of haemoglobin (Hb) function often play a key role in regulating an adaptive response to high-altitude hypoxia^14^,^15^,^16^. For example, the haemoglobin concentration of Andean individuals who are native to different elevations is higher than those of Tibetan and sea-level residents^17^,^18^. In addition, improved oxygen (O_2_) loading and unloading are physiological marks of high-altitude adaptation^19^. The O_2_-loading capacity is determined by the Hb-O_2_ affinity. An increased Hb-O_2_ affinity helps maintain pulmonary O_2_ loading at a sufficient to maximal level of tissue oxygenation under conditions of extreme hypoxia^20^. Many highland animals have haemoglobin with high O_2_ affinity, such as alpacas^21^, Andean geese^22^, bar-headed geese^23^,^24^ and deer mice^25^,^26^.

Although many studies have focused on highland humans and animals, the genetic mechanisms underlying the adaptation of domesticated animals to the Tibetan Plateau have been rarely studied. In this study, a genome-wide investigation of the adaptation of Tibetan sheep to the high-altitude plateau was conducted using the Illumina Ovine SNP50K Bead Chip assay. We performed whole-genome SNP sweeps to study the adaptive evolution of high-altitude sheep by analyzing seven breeds. We identified candidate genes using selective sweep mapping and revealed potential genetic mechanisms of high-altitude adaptation in sheep compared with other species. We also more deeply analyzed gene evolution by investigating genes and networks as well as the genetic diversity of *EPAS1*.

## Results

### Population structure

**Figure 1:**
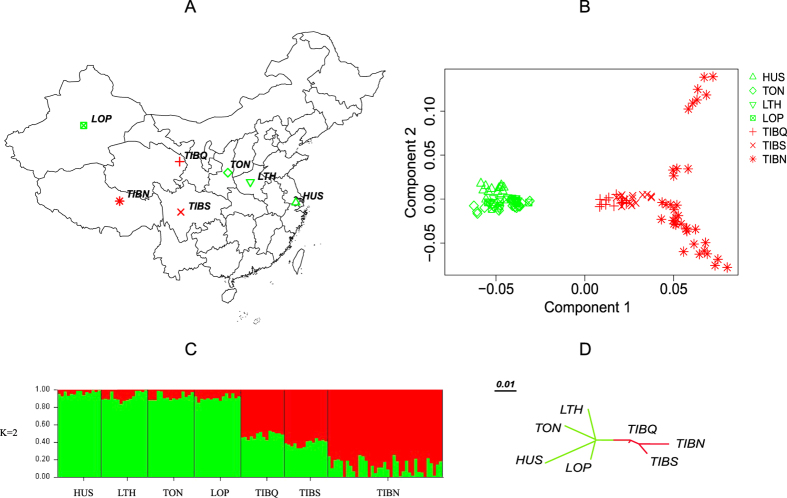
Description of the location and genetic relationships of samples. (**A**) Sampling and habitats of the seven Tibetan sheep breeds. The map was created using the R package ‘maptools’, version: 0.8–37, URL: http://r-forge.r-project.org/projects/maptools/. (**B**) Plots for the first (Component 1) and second (Component 2) dimensions revealed the clustering of all individuals. (**C**) Genome-wide admixtures inferred by STRUCTURE 2.3.4. The results from *K* = 2 are shown; (**D**) Neighbour-joining (NJ) phylogenetic tree for the seven breeds based on pairwise *F*_ST_. Each population is represented by a different symbol and colour label: high-altitude breeds are indicated in red, and low-altitude breeds are indicated in blue. The abbreviations for the seven breeds are shown in Supplemental Table S1.

To assess the relationships between the animals and breeds under investigation, we applied multidimensional scaling (MDS) to analyze 122 individuals genotyped at 20,632 autosomal SNPs. The individuals were representative of seven native Chinese sheep breeds: Hu sheep (HUS), Tong sheep (TON), Large-tailed Han sheep (LTH), Lop sheep (LOP), Tibetan sheep of the Qinghai (TIBQ), Sichuan (TIBS) and Nagqu (TIBN). (Figure 1A, Supplemental Table S1). The first dimension (Component 1) separated domestic individuals into two broad non-overlapping clusters (Fig. 1B). The clusters corresponded well with the geographic origin of each breed within the cluster. Our cluster results showed that the TIBQ, TIBS and TIBN breeds were grouped together as the highland group (G1) and that the HUS, TON, LTH and LOP breeds belonged to the lowland group (G2). To explore further the relationships obtained using the MDS approach, we performed a population structure analysis using the program STRUCTURE 2.3.4^27^ to obtain a model-based unsupervised hierarchical clustering of the individuals arising from a user-defined number of ancestral populations (*K*). The number of populations was varied from *K* = 2 to 4, and the largest change in the log of the likelihood function (Δ*K*) was found when *K* = 2 (Fig. S1), which suggests that there is likely a small number of ancestral groups. Additionally, the individuals from low-altitude breeds appeared distinct from those sampled from high-altitude breeds (Fig. 1C, Fig. S2), which was consistent with the MDS results. To further confirm the phylogenetic relationships among the sheep breeds, a neighbour-joining tree^28^ based on the pairwise genetic distances was constructed. The tree also indicated that high-altitude (TIBQ, TIBS and TIBN) and low-altitude breeds (TON, HUS, LOP and TLH) were split into two distinct branches (Fig. 1D).

### Selection signal is correlated with high altitude

*F*_ST_, which can reveal a high coefficient of genetic differentiation, has been widely used to identify selective signals among whole-genome SNPs^29^. We evaluated the population differentiation and calculated the *F*_ST_ for each SNP between G1 and G2, ultimately identifying 464 SNPs with extremely high values (*F*_ST_ > 0.242, top 1%) from the total tested (46,355 SNPs, Supplemental Table S2). Moreover, we used *F*_*ST*_ outlier tests by LOSITAN^30^ to identify loci with levels of differentiation between the G1 and G2 pairs that were higher or lower than expected under a neutral model, and approximately 96% of the outlier loci were the same as the top 1% *F*_ST_ value SNPs obtained using the Genepop software^31^ (Supplemental Table S2). Moreover, 40,652 pairwise XP-EHH (Cross Population Extend Haplotype Homozygosity Test) values, which are haplotype-based parameters, were calculated for each SNP^32^. In total, 2000 unique SNPs were within the top 5% of XP-EHH values (XP-EHH > 0.415) (Supplemental Table S3). The SNPs that were identified by high *F*_ST_ and XP-EHH values produced a subset of 171 SNPs, which represented 152 candidate genes with strong signatures of selection (Supplemental Table S4). In addition, 70 SNPs with high *F*_ST_ but null XP-EHH values were included, representing 84 candidate genes (Supplemental Table S5). Finally, genome-wide polymorphisms revealed chromosomal regions that contained 236 genes with evidence of positive selection.

We performed Gene Ontology (GO) enrichment analyses on the highlighted candidate genes in Tibetan sheep populations (Table 1). These genes are involved in response to hypoxia (GO:0001666) and several other biological processes, such as cell morphogenesis involved in neuron differentiation (GO:0048667), axonogenesis (GO:0007409), tissue homeostasis (GO:0001894) and cell maturation (GO:0048469), all of which likely play roles in the local adaptation of Tibetan sheep. To further understand the functions of the candidate genes, we compared them with several genes that are considered likely to be associated with high-altitude adaptation, which led us to prioritize a set of 317 functional candidate genes (Supplemental Table S6). In our Tibetan sheep breeds, we found that the set of candidate loci contained *EPAS1, NF1, LONP1, SOD1* and *DPP4* as well as the homologous genes *PPARG* and *TGFBR3* of the priority candidate genes.

Hypoxia-inducible factors (HIFs) are transcription factors that respond to changes in the available oxygen in the cellular environment under high-altitude conditions. *EPAS1*, also known as HIF2a, is a member of the HIF family that responds to changes in available oxygen in the cellular environment under high-altitude conditions^33^. Three candidate genes, *FGFR2, NF1* and *RasGef4*, function in the Ras/ERK signalling pathway, which commonly promotes angiogenesis with the HIF pathway under hypoxia^34^ and may have been selected for this function. Ras is indirectly activated by *FGFR2*^35^, and *RasGef4*^36^,^37^ activates Rap1, which regulates the proliferation and migration of human umbilical vein endothelial cells via the ERK and Akt pathways. *NF1* is a negative regulator of the Ras signal transduction pathway^38^, and the loss of *NF1* results in the activation of the Ras signalling pathway, leading to the aberrant growth of haematopoietic cells^39^. *DYSF* and *ZEB1* are also target genes of HIFs. The genetic loss of dysferlin (encoded by *DYSF*) caused an abrogated angiogenic response to vascular endothelial growth factor^40^. A study found that *ZEB1* is critical in the regulation of the macrovascular angiogenic response but not that of microvascular angiogenesis^41^. Moreover, under hypoxic conditions, HIF-1α up-regulates the expression of proteins that induce *TWIST1* and *ZEB1*^42^,^43^. However, in different animals, different genes affecting angiogenesis have been detected, such as *DAM17*^10^, *NOS3*^4^ and *PLXNA4*^6^ in yaks, Tibetan antelope and Tibetan Mastiffs, respectively.

HIFs were discovered because of their ability to stimulate transcription of the erythropoietin (EPO) gene during hypoxia at high altitudes^43^,^44^,^45^. Here, *SOCS2* and *LONP1* were also identified as candidate selected genes associated with the primary role of HIFs in promoting the hypoxic EPO response. *SOCS2* regulates EPO-enhanced neuronal differentiation^46^ and, together with *SOCS3*, can regulate EPO signalling in response to hypoxia^47^. Lon peptidase 1 (*LONP1*) is a multifunctional ATP-dependent protease that mainly participates in mitochondrial proteolysis, is involved in heme biosynthesis^48^,^49^ and can regulate the EPO gene^50^. Under conditions of reduced O_2_ availability, HIF-1α reciprocally regulates *COX4* subunit expression by activating the transcription of the gene encoding *LONP1*^51^.

Candidate genes participating in energy metabolism have been identified regularly in animals living in high-altitude areas^4^,^10^,^11^,^52^. In Tibetan sheep, we identified a few genes associated with energy metabolism that were under positive selection. *DPP4* is a key enzyme involved in glycolysis, where it degrades GLP-1, which is powered by the hydrolysis of ATP, and plays crucial roles in maintaining glucose stability^53^,^54^,^55^. *PPARG*, another selected gene, reactivates adipogenesis and transcriptionally activates *LPP1* expression, indicating a potential role in the metabolism of phospholipids^56^,^57^. Substantial evidence has shown that mitochondrial function is altered in high-altitude adaptations^58^. A change is initiated by reduced oxygen delivery to cells during metabolism, and reactive oxygen species (ROS) are produced by hypoxic mitochondria, which stimulate the activation of HIF-1 and HIF-2^59^,^60^. *SOD1*, the anti-oxidant superoxide dismutase 1, which activates ATP production^61^ and reduces mitochondrial ROS production^62^, was identified as being under positive selective pressure in Tibetan sheep.

Hypoxia causes an influx of Ca^2+^ and an increase in intracellular Ca^2+^ concentrations by opening store-operated Ca^2+^ (SOC) channels, which results in increased cell contraction^63^. In our study, *RYR3* was found to encode the ryanodine receptor 3, a calcium channel, and showed selective signatures in a study in Tibetan Mastiffs^6^. The calcineurin genes (*PPP3R1* and *PPP2R1B*), which encode protein phosphatases, play crucial roles in regulating Ca^2+^/calmodulin. Calcineurin can maintain vascular structure and function, and vascular smooth muscle cell proliferation. Calcineurin is a unique calcium/calmodulin-regulated protein phosphatase that functions as a key mediator of the hypertrophic response of the heart. *PPP3R1* (protein phosphatase 3) and *PPP2R1B* (protein phosphatase 2) are regulatory subunits of calcineurin that are important for phosphatase activity^64^.

Tibetan sheep develop low partial oxygen pressure and are exposed to high levels of ultraviolet radiation. Another important candidate selected gene is microphthalmia-associated transcription factor (*MITF*). Hypoxia up-regulates cyclooxygenase-2, leading to the prostaglandin E(2)-mediated loss of *MITF* in cervical stromal cells^65^. In our study, *MITF*, which is associated with melanogenesis, was detected as positively selected in Tibetan sheep. *MITF* was also identified as being subject to high selective pressure in the world’s sheep breeds^66^.

*HSD11B1L, HP, TGFBR3* and *MSRB3* were also identified as candidate selected genes in Tibetan sheep. *HSD11B1*, which encodes an isoform of the enzyme 11beta-hydroxysteroid dehydrogenase, acts exclusively as an NAD-dependent dehydrogenase in activating cortisol to cortisone^67^, whose activity is down-regulated under high-altitude hypoxia conditions^68^. In Tibetan sheep, another isoform product of *HSD11B1L*, which is believed to act predominantly as an oxo-reductase using NADP(H) as a cofactor to generate cortisone^69^, was identified as a candidate selected gene; this result indicates that 11beta-HSD1a is also involved in the adaptive response to high altitudes. *TGFBR3* is associated with TGF-β, which is an HIF-responsive product that is suspected of playing a role in cancer^70^,^71^. *TGFBR3* is involved in regulating cell growth and differentiation and in inflammatory responses^72^,^73^,^74^. Haptoglobin (Hp; encoded by *HP*) is a plasma glycoprotein, the main biological function of which is to bind free Hb and prevent the loss of iron and subsequent kidney damage following intravascular haemolysis^75^. The gene encoding methionine sulfoxide reductase B3 (*MSRB3*) has been identified as a gene associated with altitude in dogs^5^, although its association with ear types has also been previously reported^76^,^77^. In the world’s sheep breeds, *MSRB3* on sheep chromosome 3 was noted to develop high selection pressure^66^.

**Figure 2:**
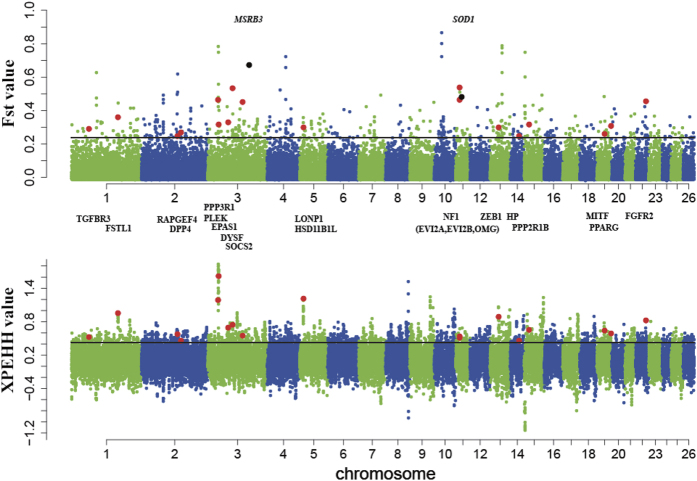
Genome-wide distribution of *F*_ST_ and XP-EHH values. Red dots represent sites showing significant signals in both the *F*_ST_ and XP-EHH approaches; black dots represent sites showing significant signal in the *F*_ST_ approach only. The symbols for candidate genes for adaptation to high-altitude hypoxia in the map are shown in bold and italics.

Some particularly extreme values for either *F*_*ST*_ or XP-EHH were also noticed (Fig. 2). For *F*_*ST*_, three genes that had undergone strongly selection were reported in previous studies^66^,^78^. *PPP1CC* (*F*_*ST*_ = 0.78, chromosome 13) is a positional candidate locus for skeletal muscle strength phenotypes^79^. *RXFP2* (*F*_*ST*_ = 0.72, chromosome 10) is a candidate gene for sheep horns^80^. *BMP2* (*F*_*ST*_ = 0.63, chromosome 13) is associated with body size traits^81^. Additionally, for XP-EHH, four genes with strong signatures of selection were detected in the highland group. *WDR92* (XP-EHH = 1.83, chromosome 3) promotes apoptosis induced by tumour necrosis factor-α (TNF-α)^82^. *PNO1* (XP-EHH = 1.62, chromosome 3) is vital to normal cell function^83^. *PARK2* (XP-EHH = 1.30, chromosome 8) is expressed primarily in the nervous system and is part of the multi-protein E3 ubiquitin ligase complex^84^. *OSR2* (XP-EHH = 1.25, chromosome 9) is a key intrinsic regulator of palatal growth and patterning^85^.

### *EPAS1* mutations and physiological associations

To further study the functions of the core factor EPAS1 in high-altitude adaption, we measured six haematological parameters in a high-altitude breed Tibetan sheep breed (TIBQ) and a low-altitude sheep breed (LTH). There were no significant differences between males and females within the breeds (Supplemental Table S7). In the high-altitude breeds, the red blood cell (RBC) count, haemoglobin (HGB) concentration, haematocrit (HCT) concentration, mean corpuscular volume (MCV) and mean corpuscular haemoglobin (MCH), but not the mean corpuscular haemoglobin concentration (MCHC), were higher or significantly higher than those of the low-altitude breeds (Supplemental Table S7). This result was similar to that found in dogs^5^. *EPAS1* polymorphisms in native Tibetan people are associated with lower haemoglobin concentrations^13^.

**Figure 3:**
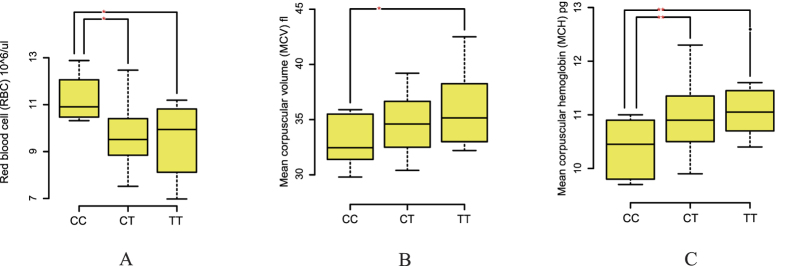
Genotype–phenotype association with the (**A**) RBC, (**B**) MCV and (**C**) MCH parameters for the 11^th^ SNP in *EPAS1* in the Tibetan breeds. *Indicates a significant difference between the genotypes; **Indicates an extremely significant difference between the genotypes.

To understand the relationship between *EPAS1* and haematological parameters, we designed 10 pairs of primers and detected 12 mutations in the exons of *EPAS1* using Sanger sequencing. Ten mutations were in the coding region, including three non-synonymous mutations, six synonymous mutations, and an AGC insert; two SNPs were in the 3′ untranslated regions (Supplemental Table S8). Chi-squared tests showed that the frequencies of the 1^st^, 2^nd^, 5^th^ and 11^th^ mutations were significantly different (P < 0.05) between the populations (Supplemental Table S9). In the Tibetan breeds, alleles of these four SNPs were tested for association with haematological parameters, and only the 11th SNP showed a significant difference. The CC genotype was associated with a significantly higher RBC count and significantly lower MCV and MCH than the TT genotype (Fig. 3).

## Discussion

Tibetan sheep are a hypoxia-tolerant species that live in an extremely inhospitable high-altitude environment, which has high ultraviolet radiation and a low partial pressure of oxygen compared with low-altitude areas^86^. In our study, we genotyped 122 sheep from seven breeds from high and low altitudes using a 50K SNP chip. SNP diversity was examined within the samples of native sheep that were the first to be domesticated by humans. This was the first study to characterize the genetic polymorphisms and evolution of Tibetan sheep.

The examination of a greater number of SNPs allowed STRUCTURE and MDS to robustly detect a distinct geographic pattern within the breeds genotyped. In our study, the seven Chinese indigenous breeds could be divided into two groups (G1 and G2) based on their genetic structure. G1 included Tibetan sheep (TIBQ, TIBS and TIBN) that live in the Qinghai Tibet plateau mountainous area, with an altitude over 3000 m, whereas Mongolian sheep (HUS, TON, LTH and LOP), which live at an altitude under 1000 m on the plain and basin, were clustered in G2 (Fig. 1A).

To detect the potential mechanisms of adaptation to high altitudes and ultraviolet radiation, we searched for SNPs that showed evidence of specific selection between the Tibetan sheep breeds that live at high altitude and the breeds that live at low altitude by using the *F*_ST_ and XP-EHH methods, which both have good statistical power for detecting selection signals among genome-wide SNP genotypes, although the sample sizes are limited^87^,^88^. Genome-wide polymorphisms revealed chromosomal regions that contained 236 genes with evidence of positive selection. Their GO annotation showed that the three genes examined (*CRYAA, EPAS1* and *LONP1*) are involved the hypoxia response (GO:0001666, P _Bonferroni_ < 0.05). Additionally, neuron development (GO:0048667, GO:0007409, P _Bonferroni_ < 0.05) was affected by the highland environment. Moreover, we found 13 genes among the candidate genes (*NF1, PPP3R1, HSD11B1L, ZEB1, SOCS2, DYSF, PPP2R1B, MITF, RAPGEF4, MSRB3, TGFBR3, HP* and *DPP4*) that have other plausible biological functions associated with high-altitude adaptation (Fig. 2).

Several of the selected genes in Tibetan sheep have also been identified in other species. For example, *EPAS1* was selected in both Tibetan humans^12^ and Tibetan Mastiffs^5^,^6^. *MSRB3* was selected in Tibetan Mastiffs and modern sheep^5^,^66^. Moreover, *MITF* showed the strongest balancing selection signal among the world’s sheep breeds^66^. The major function of *MITF*, as the target of novel melanoma amplification^89^, is melanocyte differentiation, which might play a critical role in melanogenesis^90^. These results indicate that *MITF* might be associated with adaptation to high ultraviolet levels at high altitudes.

Other candidate genes demonstrating signals of selection did not overlap among different high-altitude species, although they were inferred to have similar functions. In this study, *DPP4*^53^,^54^,^55^, *PPARG*^58^,^59^ and *SOD1*^61^ were associated with energy metabolism. *FGFR2*^35^, *NF1*^38^ and *RasGef4*^37^,^38^ were found in the Ras/ERK signalling pathway, which plays a role in hypoxia, along with the HIF pathway, by promoting angiogenesis. Additionally, *DYSF* and *ZEB1* play important roles in angiogenesis. *SOCS2*^46^,^47^ and *LONP*^51^ were also identified as candidate selected genes associated with the primary role of HIFs in promoting the EPO response under hypoxia. *PPP3R1* and *PPP2R1B*^64^ play crucial roles in regulating Ca^2+^/calmodulin. *HP*^75^ is a plasma glycoprotein whose main biological function is to bind free haemoglobin and prevent the loss of iron. *HSD11B1L*^67^ is believed to predominantly act *in vivo* as an oxo-reductase using NADP(H) as a cofactor to generate cortisone.

**Figure 4:**
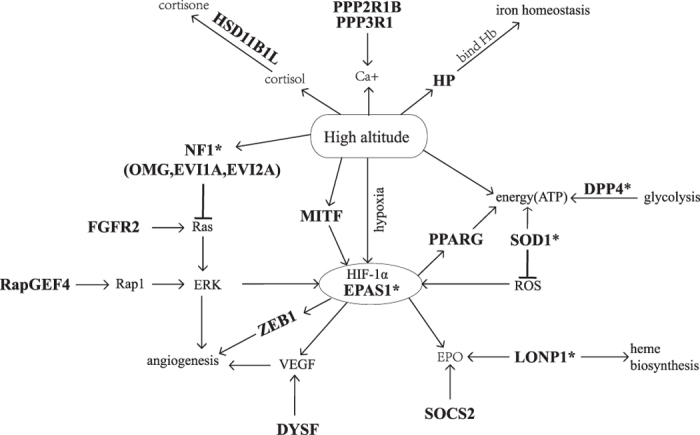
Complexity of plausible pathways of positively selected genes in high-altitude adaptation in Tibetan sheep. The symbols for candidate genes for adaptation to high-altitude in the map are shown in bold. Candidate loci marked with an asterisk (*) represent the hypoxia priority candidate genes. Candidate selected genes are associated with hypoxia, energy metabolism, angiogenesis, Ca^2+^ metabolism, generating cortisone, erythropoietin and iron homeostasis under high-altitude conditions.

Furthermore, we constructed a complex network of plausible pathways of positively selected genes for high-altitude adaptation in Tibetan sheep based on the functions of these genes, which are involved in hypoxia, energy metabolism, angiogenesis, Ca^2+^ metabolism, cortisone generation, erythropoietin and iron homeostasis under high-altitude conditions (Fig. 4). Network analysis demonstrated that most of the scanned candidate hypoxia-response genes are regulated by the HIF signalling pathway, thus indicating the vital role that *EPAS1* plays in high-altitude adaptation in Tibetan sheep. This result is similar to that observed in dogs^5^,^6^. Processes mediated by these HIFs have been detected in both humans and Tibetan Mastiffs in response to high-altitude conditions and include iron homeostasis^91^, erythropoiesis^44^, vascular permeability^92^,^93^, glycolytic protein angiogenesis and metabolism^94^,^95^,^96^,^97^. This result indicates that different high-altitude animals have similar signatures of adaptive evolution in response to the environment.

Interestingly, *EPAS1* was selected in Tibetans, Tibetan Mastiffs and Tibetan sheep. This indicates that parallel evolution at the molecular level may exist among these species. With the assistance of their human companions, dogs dispersed into new environments as human civilization expanded during the Paleolithic period^98^, a finding that is consistent with the time at which dogs were domesticated. Humans settled on highlands approximately 25,000 years ago^99^. The domestication of sheep occurred during the Neolithic period, approximately 9000 years ago^100^, in nearby regions in Iran and in a region of northern Iraq^101^.

*EPAS1* plays an important role in high-altitude adaption in humans and dogs^5^,^13^. Tibetans and Tibetan Mastiffs display higher blood flow for oxygen delivery^5^,^102^, and this gene is related to the haemoglobin concentration in Andeans^103^ and Tibetans^13^,^104^,^105^,^106^. Therefore, we propose that similar mechanisms may have facilitated sheep adaptation to high-altitude hypoxia. In this study, we also found that *EPAS1* had a high positive selection signal in Tibetan sheep breed. One limitation of this study is its relatively small sample size; consequently, the conclusions cannot be extrapolated to all sheep populations. In this study, we examined 12 mutations in the exons of *EPAS1* in detail. However, only four SNPs with different allele frequencies between high- and low-altitude breeds were identified. Mutations in the *EPAS1* coding region and phenotypes are not associated with haematologic parameters; however, in the 3′ untranslated regions, one SNP did show an association: the CC genotype was associated with a significantly higher RBC count and significantly lower MCV and MCH compared to the TT genotype in Tibetan sheep. MCV was found to differ among humans living at high altitudes and at sea level, with significantly higher values associated with humans living at higher altitudes^107^. These results indicate that increased MCV and MCH are associated with the enhanced ability to carry oxygen under conditions of high-altitude hypoxia. Consistent with this observation, reports of *EPAS1* mutations in human patients have been exclusively associated with haematological phenotypes, especially alterations in MCH^104^. Our results indicate that the allele targeted by selection likely confers a functionally relevant adaptation to the hypoxic environment at high altitudes, although the precise physiological mechanism remains to be discovered.

## Methods

### Ethics Statement

All animals were handled according to the Guidelines for the Biological Studies Animal Care and Use Committee, People’s Republic of China. Animal experiments were approved by the Animal Ethics Committee of the Institute of Animal Sciences of Chinese Academy of Agricultural Sciences.

### Genotyping and quality control

For the six Chinese sheep breed data set (Supplemental Table S1), blood samples were collected from 12 HUS, 15 TON, 15 LTH, 15 LOP, 14 TIBQ and 14 TIBS sheep. These animals were recently used in the registration and recording system of the National Center for Preservation and Utilization of Genetic Resources of Domestic Animals, National Animal Husbandry Service, Beijing, China. All experimental and surgical procedures were approved by the Biological Studies Animal Care and Use Committee, People’s Republic of China. In total, 87 DNA samples were extracted by the IQS DNA extraction method and were genotyped using the Illumina Ovine SNP 50K Bead Chip assay system at Capital Bio Corporation (Beijing, China). The second data set used in this study came from the Ovine HapMap project of the International Sheep Genomics Consortium and is publicly available at http://www.sheephapmap.org. The Chinese TIBN breed including 37 individuals was selected in the current study (Supplemental Table S1). We combined the two data sets and obtained 122 individuals and 49,034 SNPs. SNPs that could not pass the following three criteria were removed: (1) SNPs with a minor allele frequency >0.01; (2) maximum per-SNP missing rate < 0.05; and (3) SNPs on chromosome X. After quality control, there were 122 subjects and 46,355 SNPs in the analyzed dataset. Nucleotide diversity π values (Supplemental Table S1) were calculated for each breed using the Bio::PopGen::Statistics package in BioPerl (v1.6.1)^108^. Among the 7 breeds, LOP showed the highest diversity (π = 7.84 × 10^−6^), and TIBN had the lowest diversity (π = 7.32 × 10^−6^).

### Population analysis

Before analysis, all the SNPs were pruned using the indep-pairwise option, with a non-overlapped window size of 25 SNPs, a step of five SNPs, and a pairwise r^2^ threshold of 0.05, resulting in 20,632 independent SNP markers. MDS was performed using PLINK 1.07^109^. Pairwise identity-by-state distances were calculated among all the individuals using the 20,632 independent SNP markers, and MDS components were obtained using the mds-plot option, based on the identity-by-state matrix. Population structure was evaluated using STRUCTURE 2.3.4^27^. All animals were analyzed in triplicate for *K* = 2–4. All analyses were performed with a burn-in length of 20,000, followed by 30,000 MCMC (Markov Chain Monte Carlo) replications for each *K*-value. The solutions for each *K* were visualized using DISTRUCT 1.1^110^.

### Priority candidates for adaptation to high-altitude hypoxia

Except for the strongest, clearest selective signals, it is difficult to confidently distinguish true signals from false positives using population genetic data alone^111^. Thus, we generated a set of priority functional candidate loci; 247 genes were obtained from Simonson *et al*.^12^, and we searched for 128 genes using his approach, from GO categories, which were associated with hypoxia using the *Bos taurus* background (http://amigo.geneontology.org/amigo). The resulting 317 functional candidate loci, including those from *Homo sapiens* and *B. taurus*, are listed in Supplemental Table S6.

### Statistical analysis

Before statistical analysis, TIBQ, TIBS and TIBN were clustered into G1, whereas TON, HUS, LOP and TLH were clustered into G2. *F*_ST_ values per-SNP were calculated using Genepop 4.2.2^31^. The formulae proposed by Weir and Cockerham^29^ were used to analyze a single locus. The *F*_ST_ value can theoretically range from 0 to 1. Next, the outlier tests were implemented in LOSITAN, which was run using 50,000 simulations, a ‘neutral’ mean *F*_*ST*_, confidence intervals of 95% and a false discovery rate of 0.1, using the infinite alleles model^30^.

To determine if there was selection in G1, we computed the XP-EHH values using haplotype information in the xpehh program from http://hgdp.uchicago.edu/Software/. The XP-EHH derives from the idea of extended haplotype homozygosity (EHH), which is defined as the probability that two randomly chosen extended haplotypes carrying a given core haplotype are homozygous^112^. XP-EHH is used to test whether the site is homozygous in one population but polymorphic in another population through comparison of EHH scores for one core SNP between two populations^112^. A negative XP-EHH score indicates that selection occurred in the reference population, whereas a positive XP-EHH score indicates that selection occurred in the observed population.

Haplotypes were estimated with fastphase 1.4^113^. We used population label information to estimate the phased haplotype background and the following options for each chromosome: -Ku40 -Kl10 -Ki10. Candidate region annotations were obtained from Ovis 3.1 of the sheep genome from NCBI (ftp://ftp.ncbi.nih.gov/genomes/Ovis_aries/protein/). To perform functional enrichment of the candidate genes, which was required by the ClueGO plugin of Cytoscape 3.2.1^114^ using Symbol ID as input parameters, the background organism selected *B. taurus*. P values less than 0.05 after Bonferroni correction for multiple testing were considered statistically significant.

### Physiological measurement and association analysis

To verify the accuracy of the experiments, we also sampled Tibetan and Mongolian sheep from different altitudes. Jugular venous blood samples from 30 one-year-old TIBQ sheep (16 males and 14 females) that lived at least 3000 m high in Gansu Province and 31 one-year-old LTH sheep (16 males and 15 females) that lived no higher than 100 m in altitude in Beijing were collected to measure the six haematological parameters: RBC, HGB, HCT, MCV, MCH, and MCHC using a BC-2800Vet Auto Hematology Analyzer (Mindray Co., Ltd, Shenzhen, China). For the association analysis, 10 primer pairs were designed (Supplemental Table S10), and 19 *EPAS1* exons were amplified. Twelve mutations were detected and genotyped by traditional Sanger sequencing. We next used the general linear model with sex as a covariate in SPSS 20.0 to determine the association between haematological parameters and each genotype in the Tibetan breeds. The least square difference test was used for post-hoc analyses.

## Additional Information

**How to cite this article**: Wei, C. *et al*. Genome-wide analysis reveals adaptation to high altitudes in Tibetan sheep. *Sci. Rep.* **6**, 26770; doi: 10.1038/srep26770 (2016).

## Supplementary Material

Supplementary Information

## Figures and Tables

**Table 1:** GO terms enriched with candidate genes for high-altitude adaptation in Tibetan sheep.

**ID**	**Term**	**P value**	**P**_**Bonferroni**_ **value**	**Associated genes**
GO:0048667	cell morphogenesis involved in neuron differentiation	6.60E-05	0.0017	*CHN1, CNP, EPHA8, FGFR2, NUMBL, OMG, SOD1, SPTBN4, TBR1*
GO:0022829	wide pore channel activity	7.23E-05	0.0017	*GJA3, GJB2, VDAC2*
GO:0007409	axonogenesis	1.26E-04	0.0029	*CHN1, CNP, EPHA8, FGFR2, NUMBL, OMG, SPTBN4, TBR1*
GO:0016877	ligase activity, forming carbon-sulfur bonds	5.51E-04	0.0121	*ACLY, ACSL6, SLC27A2*
GO:0005681	spliceosomal complex	6.95E-04	0.0146	*DHX38, HNRNPK, RALY, SMNDC1, TXNL4B, U2AF1*
GO:0001666	response to hypoxia	2.71E-02	0.0271	*EPAS1,CRYAA, LONP1*
GO:0001894	tissue homeostasis	2.71E-02	0.0271	*EPAS1, PTHLH, SOD1*
GO:0048469	cell maturation	2.71E-02	0.0271	*EPAS1, EPHA8, PPARG*
