# Supplementary material for: Genome-wide analysis reveals adaptation to high altitudes in Tibetan sheep
Source: Sci Rep. 2016 May 27;6:26770. doi: 10.1038/srep26770 (PMC4882523; doi:10.1038/srep26770)
Supplement: Supplementary Information [file srep26770-s1.pdf]

Title

Genome-wide analysis reveals adaptation to high-altitude in Tibetan sheep

Author list

Caihong Wei, Huihua Wang, Gang Liu, Fuping Zhao, James W. Kijas, Youji Ma, Jian Lu, Li Zhang, Jiaxue Cao, Mingming Wu, Guangkai Wang, Ruizao Liu, Zhen Liu, Shuzhen Zhang, Chousheng Liu, Lixin Du

Supplementary Information

Table S1. The details of 7 sheep populations (breeds)
Table S2. A total of 464 SNPs which were within the top 1% of F_{ST} value distribution
Table S3. A total of 2001 SNPs which were within the top 5% of XPEHH value distribution
Table S4. Annotation of a SNP which both have high F_{ST} and XPEHH value
Table S5. Annotation of a SNP which just have high F_{ST} value
Table S6. List of a priori function candidate genes
Table S7. Six hematologic parameters information for TIB and LTH breeds
Table S8. Summary of variation consequences in EPAS1 gene
Table S9. The MAF and X^2 for each locus of TIB and LTH breed
Table S10. PCR primers used in this study

Figure legends

Figure S1. Mean L(K) (±SD) for each K value.
Figure S2. Structure analysis on all the individuals with K=2-4. The abbreviations for the 7 China native breeds are shown in Supplemental table S1

Table S1. The details of 7 sheep populations (breeds)

Code	Breed name	Location	Altitude (m)	n	SNP no.	Nucleotide diversity π ($\times 10^{-6}$)
HUS	Hu sheep	Jiangsu	50	12	43,618	7.61
TON	Tong sheep	Shaanxi	850	15	44,642	7.66
LTH	Large-tailed Han sheep	Shandong	50	15	44,536	7.65
LOP	Lop sheep	Xinjiang	887	15	45,349	7.84
TIBQ	Plateau-type Tibetan	Qinghai	3500	14	44,415	7.64
TIBS	Valley-type Tibetan	Sichuan	3500	14	43,782	7.51
TIBN#	Tibetan sheep	Nagqu	4500	37	44,948	7.32

Ovine HapMap project data

Table S2. A total of 464 SNPs which were within the top 1% of F_{ST} value distribution

SNP-ID	Chromosome	Position	F_{ST} (top 1%)	*p-value* (Simul F_{ST}<sample F_{ST})	FDR
OAR10_29511510.1	10	29476678	0.866	1.0000	outlier
OAR10_29469450.1	10	29436435	0.801	1.0000	outlier
s27419.1	13	48968332	0.788	1.0000	outlier
s05744.1	3	40080488	0.782	1.0000	outlier
OAR13_52482285.1	13	49006951	0.775	1.0000	outlier
s53921.1	3	40294935	0.749	1.0000	outlier
OAR15_3091174.1	15	3706790	0.748	1.0000	outlier
OAR13_52630089.1	13	49140884	0.745	1.0000	outlier
OAR4_72720594.1	4	68754789	0.722	1.0000	outlier
OAR10_29538398.1	10	29502667	0.721	1.0000	outlier
OAR3_165009241.1	3	154213690	0.671	1.0000	outlier
OAR4_72568832.1	4	68601186	0.657	1.0000	outlier
OAR13_51727898.1	13	48493120	0.626	1.0000	outlier
OAR1_100921673.1	1	94993712	0.625	1.0000	outlier
OAR2_143916009.1	2	135304995	0.619	1.0000	outlier
OAR15_3236575.1	15	3875564	0.600	1.0000	outlier
s51799.1	3	40441564	0.556	1.0000	outlier
OAR11_18701428.1	11	18325488	0.538	1.0000	outlier
OAR11_18711856.1	11	18335747	0.538	1.0000	outlier
s64982.1	3	93725974	0.534	1.0000	outlier
s67952.1	3	93522006	0.533	1.0000	outlier
OAR4_51489408.1	4	48669962	0.523	1.0000	outlier
OAR11_18815864.1	11	18433474	0.510	1.0000	outlier
OAR2_143949087.1	2	135337045	0.509	1.0000	outlier
OAR13_51852034.1	13	48623826	0.494	1.0000	outlier
OAR2_143068480.1	2	134598112	0.494	1.0000	outlier
s55494.1	7	82545552	0.493	1.0000	outlier
OAR2_86948508.1	2	81785728	0.492	1.0000	outlier
OAR15_78598295.1	15	72520678	0.490	1.0000	outlier
s74549.1	19	29047903	0.485	1.0000	outlier
OAR11_27654920.1	11	26421905	0.480	1.0000	outlier
OAR3_43027143.1	3	40285089	0.479	1.0000	outlier
OAR1_101357689.1	1	95153594	0.474	1.0000	outlier
OAR3_43817056.1	3	40956953	0.467	1.0000	outlier
OAR4_73050615.1	4	69082247	0.466	1.0000	outlier
s08514.1	3	39851694	0.464	1.0000	outlier
OAR11_18823250.1	11	18440783	0.464	1.0000	outlier
OAR2_87089443.1	2	81887124	0.464	1.0000	outlier
OAR22_45509727.1	22	40482146	0.456	1.0000	outlier
OAR3_43504420.1	3	40657958	0.453	1.0000	outlier
OAR3_43472281.1	3	40625901	0.451	1.0000	outlier

s71169.1	3	129759745	0.451	1.0000	outlier
OAR9_99601991.1	9	93576726	0.447	1.0000	outlier
OAR13_51134025_X.1	13	47855248	0.446	1.0000	outlier
OAR13_51167861.1	13	47875474	0.446	1.0000	outlier
s55347.1	1	183336939	0.443	1.0000	outlier
s65195.1	2	81853228	0.442	1.0000	outlier
s03078.1	13	33058560	0.440	1.0000	outlier
s12519.1	8	56125455	0.433	1.0000	outlier
OAR15_37466891.1	15	35619819	0.432	1.0000	outlier
OAR1_100874172.1	1	94940617	0.430	1.0000	outlier
OAR2_239847244.1	2	227091775	0.427	0.9999	outlier
OAR13_30121681.1	13	27225608	0.426	1.0000	outlier
OAR15_3164045.1	15	3805470	0.426	1.0000	outlier
OAR15_2886961.1	15	3462742	0.425	1.0000	outlier
OAR3_79206128.1	3	74982457	0.422	1.0000	outlier
OAR3_42907435.1	3	40163832	0.422	1.0000	outlier
s19503.1	22	36806429	0.420	1.0000	outlier
s14577.1	7	26755264	0.420	1.0000	outlier
s38150.1	12	29228207	0.419	1.0000	outlier
OAR1_264734445.1	1	245155764	0.413	1.0000	outlier
OAR2_193444466_X.1	2	182607165	0.413	1.0000	outlier
OAR1_248957744.1	1	230972639	0.412	1.0000	outlier
s31340.1	15	72536427	0.411	1.0000	outlier
OAR3_127924329_X.1	3	120136049	0.410	1.0000	outlier
OAR4_93067608.1	4	87649810	0.409	1.0000	outlier
OAR20_9311938.1	20	9187047	0.408	1.0000	outlier
s27209.1	12	68444880	0.405	1.0000	outlier
OAR6_67376317.1	6	61138606	0.405	1.0000	outlier
OAR15_68933568.1	15	63386447	0.402	1.0000	outlier
OAR3_44507744.1	3	41591060	0.401	1.0000	outlier
OAR9_38221892.1	9	36285902	0.398	1.0000	outlier
OAR2_193238074.1	2	182395047	0.396	1.0000	outlier
OAR6_92321965.1	6	84350628	0.392	1.0000	outlier
OARUn.1355_45411.1	15	3575948	0.387	1.0000	outlier
OAR2_142924312.1	2	134460137	0.379	1.0000	outlier
OAR3_98710538.1	3	93004742	0.378	1.0000	outlier
s61354.1	5	90586440	0.378	1.0000	outlier
OAR21_50378541.1	21	45368906	0.378	1.0000	outlier
OARUn.1355_20915.1	15	3546667	0.378	0.9999	outlier
OAR20_15759478.1	20	14939837	0.377	1.0000	outlier
s71975.1	14	16349731	0.375	1.0000	outlier
OAR2_61008309.1	2	56714900	0.371	0.9999	outlier
250506CS3900140500001_312.1	23	26298017	0.366	1.0000	outlier
OAR21_28243839.1	21	25299666	0.364	1.0000	outlier

s58920.1	17	52092174	0.364	1.0000	outlier
OAR10_36999163.1	10	36238012	0.363	1.0000	outlier
OAR4_10748026.1	4	10609123	0.362	1.0000	outlier
s37576.1	19	59099916	0.362	1.0000	outlier
s19461.1	22	39739539	0.362	0.9999	outlier
s26969.1	10	35629123	0.361	1.0000	outlier
OAR26_30396501.1	26	26301170	0.361	1.0000	outlier
OAR13_87428154.1	13	81349148	0.361	1.0000	outlier
OAR1_197916874.1	1	183420073	0.360	1.0000	outlier
OAR7_63745942.1	7	57816492	0.359	1.0000	outlier
s14938.1	9	795580	0.358	1.0000	outlier
s56763.1	5	15912332	0.357	1.0000	outlier
OAR5_87050329.1	5	79186927	0.353	1.0000	outlier
OAR15_2999185.1	15	3499482	0.353	1.0000	outlier
OAR10_37157671.1	10	36385864	0.350	1.0000	outlier
s11336.1	4	87572495	0.349	1.0000	outlier
OAR2_193298829.1	2	182455753	0.349	1.0000	outlier
OAR10_36395007.1	10	35665391	0.348	1.0000	outlier
OAR3_200888015.1	3	186673626	0.347	1.0000	outlier
s40780.1	2	9434535	0.346	1.0000	outlier
s42972.1	1	261414809	0.346	1.0000	outlier
OAR3_126195855.1	3	118325231	0.345	1.0000	outlier
OAR16_28431987.1	16	26210763	0.345	1.0000	outlier
OAR9_4919393.1	9	5044855	0.345	1.0000	outlier
s48402.1	2	60634939	0.345	1.0000	outlier
OAR2_60972128.1	2	56679411	0.343	1.0000	outlier
s45108.1	1	257631364	0.343	1.0000	outlier
OAR4_80605717.1	4	76007878	0.338	1.0000	outlier
OAR13_2388727.1	13	1861848	0.338	0.9998	outlier
s51138.1	13	47654121	0.338	1.0000	outlier
OAR4_10443301.1	4	10281638	0.337	1.0000	outlier
s39564.1	13	43797381	0.337	1.0000	outlier
s40281.1	5	95197731	0.336	1.0000	outlier
OAR3_200125923.1	3	185881136	0.336	1.0000	outlier
OAR5_50467325.1	5	46381328	0.335	1.0000	outlier
OAR10_36819129.1	10	36064927	0.335	1.0000	outlier
OAR7_63342726.1	7	57353770	0.335	1.0000	outlier
s48664.1	3	138528430	0.335	1.0000	outlier
OAR1_107504871.1	1	100185774	0.335	1.0000	outlier
s25992.1	3	136237659	0.335	1.0000	outlier
OAR9_55485114.1	9	53015370	0.335	1.0000	outlier
OAR10_28697870.1	10	28685295	0.335	0.9998	outlier
OAR19_33605872.1	19	31927501	0.335	1.0000	outlier
OAR9_82428531.1	9	77756891	0.333	1.0000	outlier

OAR4_91329005.1	4	85930010	0.332	0.9998	outlier
s23936.1	1	114785821	0.332	1.0000	outlier
DU299931_445.1	2	81407809	0.332	0.9999	outlier
OAR15_53721488.1	15	49215124	0.332	1.0000	outlier
s66575.1	4	47439380	0.332	0.9998	outlier
OAR19_14489436.1	19	13918425	0.332	1.0000	outlier
DU223332_376.1	2	59569826	0.331	1.0000	outlier
s58048.1	2	52441926	0.330	1.0000	outlier
OAR22_34323192.1	22	29843623	0.330	1.0000	outlier
OAR7_63848145.1	7	57915106	0.330	0.9998	outlier
OAR8_60234503.1	8	56216655	0.330	1.0000	outlier
OAR3_82409790.1	3	77879156	0.329	1.0000	outlier
OAR20_4842571.1	20	4835005	0.329	1.0000	outlier
OAR1_130003540.1	1	119845352	0.328	1.0000	outlier
s54574.1	13	6202204	0.327	1.0000	outlier
s67679.1	5	16017146	0.325	1.0000	outlier
s12884.1	13	62857560	0.324	1.0000	outlier
OAR5_80804886.1	5	73554040	0.322	1.0000	outlier
s32443.1	1	115427224	0.322	1.0000	outlier
s31325.1	2	182097199	0.322	1.0000	outlier
OAR1_248968063.1	1	230981011	0.321	1.0000	outlier
s07580.1	5	46208843	0.320	1.0000	outlier
OAR5_87409839_X.1	5	79542631	0.320	1.0000	outlier
s60101.1	3	186540552	0.319	1.0000	outlier
OAR8_51076568.1	8	47596948	0.319	0.9997	outlier
s04315.1	3	136258983	0.318	1.0000	outlier
OAR4_93024080.1	4	87602738	0.318	1.0000	outlier
OAR2_128835133.1	2	120501335	0.318	0.9997	outlier
OAR2_86617792.1	2	81452694	0.318	0.9999	outlier
OAR3_42729807.1	3	39986491	0.317	1.0000	outlier
OAR2_31412377.1	2	30492514	0.317	0.9998	outlier
OAR4_73786063.1	4	69811362	0.317	0.9998	outlier
s44167.1	2	243153063	0.316	1.0000	outlier
s46185.1	13	78420224	0.316	1.0000	outlier
OAR10_28087786.1	10	28071590	0.315	1.0000	outlier
OAR15_22404265.1	15	21482600	0.315	0.9994	outlier
s34725.1	2	13129990	0.314	1.0000	outlier
OAR4_25295648.1	4	24132337	0.313	0.9994	outlier
OAR7_47161420.1	7	42551974	0.313	0.9994	outlier
s66483.1	5	46430611	0.313	1.0000	outlier
s41913.1	1	2870378	0.312	1.0000	outlier
OAR3_43559179_X.1	3	40713016	0.312	0.9994	outlier
s49062.1	18	46100361	0.311	0.9994	outlier
OAR3_124838365.1	3	116944852	0.311	1.0000	outlier

OAR17_67295616.1	17	61733069	0.310	1.0000	outlier
s67316.1	22	36740014	0.310	1.0000	outlier
DU177621_558.1	19	53781872	0.309	1.0000	outlier
s05344.1	23	26312088	0.309	1.0000	outlier
OAR3_195730138.1	3	181711513	0.307	0.9998	outlier
OAR12_37060968.1	12	33067355	0.307	1.0000	outlier
s08464.1	24	35991517	0.307	0.9998	outlier
OAR19_60184770.1	19	56634864	0.307	1.0000	outlier
s60112.1	2	68855701	0.305	1.0000	outlier
s54559.1	13	52129026	0.305	1.0000	outlier
OAR3_165050963.1	3	154252449	0.305	1.0000	outlier
OAR4_9681181_X.1	4	9567863	0.305	1.0000	outlier
s24597.1	14	49053815	0.304	1.0000	outlier
OAR3_45905654.1	3	42874992	0.303	0.9999	outlier
s30783.1	9	28233297	0.303	1.0000	outlier
OAR2_55853730.1	2	52254402	0.303	1.0000	outlier
OAR18_27752153.1	18	26754749	0.303	1.0000	outlier
s50255.1	3	220327385	0.301	1.0000	outlier
OAR6_15846520.1	6	13261000	0.300	1.0000	outlier
s19468.1	10	30993691	0.300	1.0000	outlier
s07332.1	5	16209393	0.300	1.0000	outlier
s69458.1	22	40594059	0.300	1.0000	outlier
s01490.1	3	180726856	0.299	0.9995	outlier
s00950.1	2	81689824	0.299	1.0000	outlier
s66464.1	10	35981007	0.299	1.0000	outlier
OAR10_36746264.1	10	35992688	0.299	1.0000	outlier
OAR2_156184412.1	2	147210213	0.299	1.0000	outlier
OAR3_126212728.1	3	118343013	0.298	1.0000	outlier
s58543.1	13	12265286	0.298	1.0000	outlier
OAR13_36331465.1	13	32978582	0.298	0.9997	outlier
s32322.1	1	96005302	0.298	1.0000	outlier
s72346.1	25	42689823	0.298	0.9992	outlier
OAR25_12256905.1	25	12080800	0.297	1.0000	outlier
OAR12_72924306.1	12	66252130	0.297	0.9995	outlier
OAR7_6367591.1	7	6512317	0.296	1.0000	outlier
OAR3_109576568.1	3	103216209	0.295	0.9992	outlier
s38680.1	5	16520048	0.295	1.0000	outlier
OAR13_63161846.1	13	58023662	0.294	1.0000	outlier
OAR1_248896910_X.1	1	230903245	0.294	1.0000	outlier
OAR2_60936578.1	2	56639600	0.293	0.9997	outlier
OAR13_70737254.1	13	65652628	0.293	0.9992	outlier
s18401.1	13	65660964	0.293	0.9992	outlier
s33002.1	5	48043887	0.293	0.9997	outlier
OAR5_52351898.1	5	48128800	0.293	0.9997	outlier

OAR9_25714308.1	9	24740849	0.293	0.9997	outlier
OAR17_44358460.1	17	41064820	0.292	1.0000	outlier
OAR17_31561091.1	17	28790576	0.292	0.9997	outlier
OAR4_73842776_X.1	4	69869475	0.291	1.0000	outlier
OAR11_6228500.1	11	6725442	0.291	1.0000	outlier
OAR18_56445082.1	18	52750500	0.291	1.0000	outlier
s10546.1	8	87520897	0.291	0.9999	outlier
OAR16_51559181.1	16	47383354	0.291	1.0000	outlier
OAR1_45270297.1	1	43681990	0.291	1.0000	outlier
s33982.1	22	44880164	0.290	1.0000	outlier
s71442.1	10	82994149	0.290	1.0000	outlier
s66349.1	1	68199001	0.289	1.0000	outlier
s35678.1	11	51495855	0.289	1.0000	outlier
s04523.1	14	13071092	0.289	1.0000	outlier
OAR3_112640501.1	3	105894007	0.289	0.9999	outlier
OAR5_110742508.1	5	101776198	0.289	1.0000	outlier
OAR4_72440076.1	4	68468908	0.288	1.0000	outlier
s63142.1	13	32868564	0.288	1.0000	outlier
OAR2_138672719.1	2	130334245	0.288	1.0000	outlier
OAR2_55861669.1	2	52266474	0.288	1.0000	outlier
s49602.1	2	248590000	0.288	1.0000	outlier
OAR3_124131226.1	3	116419981	0.288	1.0000	outlier
OAR4_51625352.1	4	48804045	0.288	1.0000	outlier
s51044.1	3	7462549	0.288	1.0000	outlier
OAR15_66701694.1	15	61349591	0.287	1.0000	outlier
s66058.1	9	32100676	0.286	0.9996	outlier
OAR3_184174208_X.1	3	171572613	0.286	1.0000	outlier
OAR22_11354622.1	22	9513923	0.285	0.9998	outlier
s66125.1	3	93671110	0.284	1.0000	outlier
OAR19_38829925.1	19	37027503	0.284	1.0000	outlier
s22470.1	2	175650501	0.284	1.0000	outlier
OAR13_46575198.1	13	43303706	0.283	1.0000	outlier
OAR5_87274003.1	5	79408583	0.282	1.0000	outlier
OAR3_195698523.1	3	181680527	0.282	1.0000	outlier
s22929.1	10	35885222	0.281	1.0000	outlier
OAR2_86819880.1	2	81666272	0.281	1.0000	outlier
OAR8_67122251.1	8	62326453	0.280	0.9995	outlier
OAR1_268122939.1	1	248275678	0.280	0.9990	outlier
OAR3_127956387_X.1	3	120178240	0.280	0.9990	outlier
OAR3_126893362.1	3	118982570	0.280	0.9992	outlier
OAR3_96170847.1	3	90549311	0.279	0.9990	outlier
OAR7_43027191.1	7	38944126	0.279	0.9990	outlier
OAR4_74539177.1	4	70467338	0.279	0.9994	outlier
OAR17_52473999.1	17	48148077	0.279	1.0000	outlier

OAR13_14410176.1	13	14295797	0.279	1.0000	outlier
OAR1_281649480.1	1	260317241	0.278	0.9994	outlier
s16895.1	5	23139577	0.278	1.0000	outlier
OAR8_67085709.1	8	62289907	0.278	1.0000	outlier
OAR3_126384508.1	3	118516904	0.278	0.9990	outlier
OAR11_3180902.1	11	3834100	0.278	0.9992	outlier
s49314.1	7	57002225	0.277	1.0000	outlier
OAR16_34243483.1	16	31525482	0.277	1.0000	outlier
OAR11_32450209.1	11	30567032	0.277	1.0000	outlier
OAR1_177805968.1	1	164847462	0.277	1.0000	outlier
OAR4_5373295.1	4	5154846	0.277	1.0000	outlier
OAR23_49635171_X.1	23	46823961	0.277	1.0000	outlier
OAR5_22783606.1	5	20089099	0.277	1.0000	outlier
OAR5_87366264.1	5	79478908	0.277	1.0000	outlier
OAR16_26522451.1	16	24393554	0.277	1.0000	outlier
s19512.1	3	76037302	0.276	0.9992	outlier
OAR2_195165011.1	2	184070090	0.276	1.0000	outlier
OAR16_27229721.1	16	25021431	0.276	1.0000	outlier
s02602.1	1	200729179	0.276	1.0000	outlier
OAR10_18066560.1	10	18955751	0.276	1.0000	outlier
s52780.1	14	13058130	0.276	1.0000	outlier
OAR1_264697118.1	1	245120011	0.276	1.0000	outlier
OAR4_37225390.1	4	35004679	0.275	1.0000	outlier
OAR15_60961014.1	15	55556375	0.274	1.0000	outlier
OAR9_81909760.1	9	77297358	0.274	0.9999	outlier
OAR3_42529367.1	3	39786723	0.274	0.9997	outlier
OAR1_47449016.1	1	45758020	0.274	1.0000	outlier
OAR14_33142743.1	14	31832814	0.274	0.9999	outlier
s71728.1	17	71871881	0.274	0.9999	outlier
OAR3_53590166.1	3	50888555	0.273	1.0000	outlier
OAR15_42233005.1	15	40231054	0.273	1.0000	outlier
s38567.1	19	7169893	0.273	1.0000	outlier
OAR19_33593848.1	19	31915771	0.273	1.0000	outlier
OAR3_99616457.1	3	93809000	0.273	0.9999	outlier
OAR1_24797111.1	1	24578785	0.273	1.0000	outlier
OAR10_68163245.1	10	65948793	0.273	1.0000	outlier
OAR2_161368305.1	2	152215629	0.273	0.9999	outlier
OAR3_124143775.1	3	116431606	0.272	1.0000	outlier
OAR6_5659766.1	6	3750660	0.272	0.9999	outlier
OAR2_142646641.1	2	134194138	0.270	0.9999	outlier
OAR10_44207570.1	10	43591931	0.270	0.9993	outlier
OAR16_14772180.1	16	13643499	0.270	0.9992	outlier
OAR22_6354636.1	22	5331079	0.269	1.0000	outlier
OAR2_60563723.1	2	56274688	0.269	0.9992	outlier

OAR2_209681146.1	2	198116050	0.269	0.9995	outlier
s29038.1	15	40156061	0.269	1.0000	outlier
OAR10_57242083.1	10	56100245	0.268	0.9995	outlier
OAR6_31012935.1	6	27322530	0.268	1.0000	outlier
OAR25_32156972.1	25	30802961	0.268	1.0000	outlier
OAR23_9974468.1	23	9065236	0.267	1.0000	outlier
s23150.1	19	44272835	0.267	0.9998	outlier
s64015.1	23	46368708	0.267	0.9995	outlier
OAR2_142882312.1	2	134417645	0.266	1.0000	outlier
OAR2_155715202.1	2	146734798	0.266	0.9991	outlier
s32813.1	7	39390045	0.266	1.0000	outlier
s25195.1	25	7392689	0.266	0.9995	outlier
OAR10_40212146.1	10	39393584	0.266	1.0000	outlier
OAR26_46921474.1	26	41364432	0.265	0.9995	outlier
OAR11_34161931.1	11	32011837	0.265	1.0000	outlier
s51112.1	2	81758958	0.265	0.9995	outlier
OAR13_51387503.1	13	48147260	0.265	0.9994	outlier
OAR16_26006299.1	16	23950271	0.265	1.0000	outlier
OAR2_144002704.1	2	135391568	0.265	1.0000	outlier
OAR3_43377195.1	3	40534487	0.264	0.9995	outlier
s32596.1	19	45555962	0.264	1.0000	outlier
OAR21_27900981.1	21	25011236	0.264	1.0000	outlier
OAR4_80559387.1	4	75968047	0.264	1.0000	outlier
OAR3_43171845.1	3	40325396	0.264	0.9987	-
OAR10_28149069.1	10	28136160	0.264	0.9995	outlier
s24197.1	13	27210961	0.264	1.0000	outlier
s28647.1	10	36317262	0.264	1.0000	outlier
OAR3_43700480.1	3	40849521	0.264	1.0000	outlier
s25289.1	4	68802676	0.264	1.0000	outlier
OAR3_152693749.1	3	142848742	0.263	1.0000	outlier
s29343.1	9	4852716	0.263	0.9993	outlier
s54635.1	3	214112448	0.263	1.0000	outlier
OAR2_173495274.1	2	163845372	0.262	0.9987	-
OAR4_75404695.1	4	71208809	0.262	1.0000	outlier
OAR7_61660987.1	7	55726352	0.262	0.9991	outlier
s21657.1	2	246962852	0.262	1.0000	outlier
OAR6_40370293.1	6	36155169	0.262	1.0000	outlier
OAR3_138331159.1	3	129685397	0.262	0.9987	-
OAR6_32950084.1	6	29117330	0.261	1.0000	outlier
OAR10_18055683.1	10	18944666	0.261	1.0000	outlier
OAR13_61547978.1	13	56574141	0.261	1.0000	outlier
OAR22_30850267.1	22	26517035	0.261	0.9989	outlier
s33957.1	11	41563065	0.261	0.9997	outlier
s32677.1	19	31833784	0.261	0.9995	outlier

OAR3_33093517.1	3	30928786	0.261	0.9987	-
s34722.1	1	119491972	0.261	0.9999	outlier
OAR3_99622529.1	3	93811641	0.261	0.9999	outlier
s28038.1	20	18995856	0.261	0.9998	outlier
s51433.1	17	12876687	0.261	1.0000	outlier
DU397126_450.1	14	38330512	0.260	0.9989	outlier
s58695.1	16	11807075	0.260	1.0000	outlier
OAR17_33260430.1	17	30423316	0.260	0.9999	outlier
OAR2_63781435.1	2	59306755	0.260	0.9995	outlier
OAR13_36256194.1	13	32901567	0.260	0.9995	outlier
OAR21_45614480.1	21	41348152	0.260	1.0000	outlier
OAR23_42222982.1	23	39828270	0.259	0.9994	outlier
OAR6_20705814.1	6	17771785	0.259	1.0000	outlier
OAR17_31102067.1	17	28339250	0.259	1.0000	outlier
DU184544_326.1	3	108058182	0.259	1.0000	outlier
OAR3_109226159.1	3	102833573	0.259	1.0000	outlier
OAR10_28665836.1	10	28653747	0.259	1.0000	outlier
OAR5_99220435.1	5	90991529	0.259	1.0000	outlier
OAR7_63814443.1	7	57875478	0.258	0.9991	outlier
OAR14_46482530.1	14	44143535	0.258	0.9991	outlier
s43948.1	14	10834307	0.257	1.0000	outlier
OAR13_89056619.1	13	83051083	0.257	1.0000	outlier
OAR13_89063022.1	13	83057828	0.257	1.0000	outlier
s10721.1	4	94145887	0.257	1.0000	outlier
s42703.1	25	17652415	0.256	0.9998	outlier
OAR2_77016276.1	2	72254024	0.256	0.9998	outlier
OAR3_136865536.1	3	128287542	0.256	0.9997	outlier
s07518.1	5	58793978	0.256	1.0000	outlier
s70839.1	3	186368226	0.256	1.0000	outlier
OAR10_18125946.1	10	19014824	0.256	1.0000	outlier
DU464218_590.1	1	183575060	0.255	0.9986	-
s57846.1	5	58686175	0.255	1.0000	outlier
OAR1_207918006.1	3	178229618	0.254	1.0000	outlier
s12781.1	11	34852341	0.254	1.0000	outlier
OAR7_63960135.1	7	58027024	0.254	0.9997	outlier
OAR1_192706983.1	1	178722562	0.254	1.0000	outlier
OAR2_19500065.1	2	19163821	0.254	1.0000	outlier
OAR22_43788746.1	22	38880694	0.253	1.0000	outlier
s24740.1	2	110941391	0.253	0.9991	outlier
OAR21_30735369.1	21	27416751	0.253	0.9984	-
OAR15_53192586.1	15	48728528	0.253	0.9997	outlier
OAR15_58554489.1	15	53412021	0.253	0.9997	outlier
OAR13_36472387.1	13	33104912	0.252	1.0000	outlier
s42597.1	3	93819638	0.252	1.0000	outlier

OAR3_200394051.1	3	186159798	0.252	0.9993	outlier
OAR17_31280438.1	17	28493407	0.251	0.9989	outlier
OAR8_60076974.1	8	56100400	0.251	1.0000	outlier
s61045.1	16	5199230	0.251	0.9983	-
s45377.1	22	45373699	0.251	1.0000	outlier
OAR10_40339520.1	10	39489315	0.251	1.0000	outlier
s29567.1	5	16203101	0.251	1.0000	outlier
s45399.1	13	34613483	0.251	1.0000	outlier
s35979.1	11	61719571	0.250	1.0000	outlier
OAR16_33435828.1	16	30720540	0.250	0.9999	outlier
OAR2_34497203.1	2	33138364	0.250	1.0000	outlier
OAR15_14742464.1	15	14639505	0.250	0.9993	outlier
OAR4_91645832.1	4	86244865	0.250	0.9989	outlier
OAR4_96284619.1	4	90707634	0.250	0.9990	outlier
OAR7_63434047.1	7	57444290	0.249	0.9989	outlier
OAR19_46690670.1	19	44350807	0.249	0.9995	outlier
OAR3_120497798.1	3	113038732	0.249	0.9983	-
OAR3_141586525.1	3	132478420	0.249	1.0000	outlier
OAR23_9959034.1	23	9048593	0.249	0.9993	outlier
OAR14_39926553.1	14	38342841	0.248	0.9983	-
OAR1_223448974.1	1	206911610	0.248	1.0000	outlier
s44712.1	2	135753568	0.248	0.9993	outlier
OAR14_39834952.1	14	38250883	0.248	1.0000	outlier
s62522.1	16	13129166	0.248	0.9999	outlier
OAR16_15286536.1	16	14040007	0.248	1.0000	outlier
OAR1_278191481.1	1	257412265	0.247	0.9995	outlier
OAR16_42321024.1	16	38952447	0.247	0.9995	outlier
s67284.1	25	40426700	0.247	0.9995	outlier
s69776.1	2	31564277	0.247	0.9987	-
OAR13_51886803.1	13	48657027	0.247	0.9993	outlier
OAR3_200934301.1	3	186702791	0.247	0.9993	outlier
OAR7_19928730.1	7	19115796	0.247	0.9986	-
s07899.1	5	20057438	0.247	1.0000	outlier
s73962.1	3	93903353	0.247	0.9983	-
s67285.1	1	185509365	0.247	1.0000	outlier
s61002.1	16	17460862	0.247	0.9997	outlier
OAR1_249572898.1	1	231602195	0.246	1.0000	outlier
s08622.1	1	251795181	0.246	0.9983	-
s07605.1	22	40971277	0.246	0.9997	outlier
OAR26_16139285.1	26	13333552	0.246	0.9993	outlier
OAR23_27062541.1	23	25919106	0.246	0.9986	-
OAR11_9228448.1	11	9643793	0.246	0.9996	outlier
s62564.1	18	19399095	0.246	0.9997	outlier
OAR7_62173993.1	7	56184926	0.246	0.9999	outlier

OAR13_52092653.1	13	48832966	0.245	1.0000	outlier
s13456.1	3	156283498	0.245	1.0000	outlier
OAR2_156740619.1	2	147804448	0.245	0.9992	outlier
OAR2_12469561.1	2	13078663	0.245	0.9987	-
OAR2_15027825.1	2	15581003	0.245	0.9997	outlier
OAR2_172701586.1	2	163070840	0.245	0.9997	outlier
OAR15_4302920.1	15	4912198	0.245	0.9997	outlier
OAR2_63274739.1	2	58896602	0.245	0.9990	outlier
OAR2_176738567_X.1	2	167096123	0.245	0.9990	outlier
OAR9_1718056.1	9	2004658	0.245	1.0000	outlier
OAR10_87300005.1	10	79986413	0.245	1.0000	outlier
s49429.1	13	48367950	0.245	1.0000	outlier
OAR3_117760271.1	3	110513933	0.244	0.9979	-
OAR6_125621422.1	6	110561891	0.244	1.0000	outlier
s06024.1	24	34985988	0.244	0.9997	outlier
s37981.1	23	46362141	0.243	1.0000	outlier
s00079.1	3	93405961	0.243	0.9997	outlier
s17708.1	1	53355068	0.243	0.9980	-
OAR13_49902903.1	13	46588897	0.243	0.9980	-
s34341.1	22	48601164	0.243	1.0000	outlier
s40624.1	25	42786734	0.243	1.0000	outlier
OAR2_40373894.1	2	38795830	0.243	0.9990	outlier
OAR6_40780034.1	6	36572809	0.243	0.9990	outlier
s26670.1	12	43278320	0.242	1.0000	outlier
OAR3_187883913.1	3	175057434	0.242	0.9984	-
s53334.1	24	12952274	0.242	1.0000	outlier
s01434.1	1	261647336	0.242	0.9982	-

Table S3. A total of 2001 SNPs which were within the top 5% of XPEHH value distribution

SNP-ID	Chromosome	Position	XPEHH value
OAR3_42907435.1	3	40163832	1.828
OAR3_43377195.1	3	40534487	1.806
OAR3_42933940.1	3	40191001	1.794
OAR3_43472281.1	3	40625901	1.747
OAR3_43027143.1	3	40285089	1.741
OAR3_43427639.1	3	40586679	1.734
s51799.1	3	40441564	1.733
OAR3_43272838.1	3	40433789	1.725
OAR3_43504420.1	3	40657958	1.716
OAR3_42867624.1	3	40124759	1.716
OAR3_43559179_X.1	3	40713016	1.703
OAR3_43171845.1	3	40325396	1.692
OAR3_43209409.1	3	40363793	1.683
OAR3_43013103.1	3	40270213	1.645
OAR3_42729807.1	3	39986491	1.618
OAR3_43700480.1	3	40849521	1.594
OAR3_43337281.1	3	40496764	1.592
s53921.1	3	40294935	1.533
s72091.1	3	40781801	1.525
s73275.1	8	83688530	1.524
OAR3_43596712.1	3	40751920	1.513
OAR3_42672294.1	3	39927660	1.400
OAR3_43708684.1	3	40855577	1.347
OAR3_43766277.1	3	40909842	1.338
OAR3_43667754.1	3	40815230	1.318
s05744.1	3	40080488	1.310
s52712.1	8	83700844	1.304
OAR9_82620139.1	9	77910235	1.252
OAR15_88156831.1	15	79062902	1.237
OAR3_43889039.1	3	41032031	1.226
OAR3_43817056.1	3	40956953	1.218
s14846.1	3	40924473	1.217
s07332.1	5	16209393	1.217
s67679.1	5	16017146	1.212
s08514.1	3	39851694	1.185
s05406.1	9	77975261	1.176
s29567.1	5	16203101	1.175
OAR9_82999383.1	9	78316151	1.157
OAR15_88030300.1	15	79137075	1.144
s52216.1	15	79041638	1.115
OAR3_43871305.1	3	41015313	1.109

OAR9_82575979.1	9	77866279	1.095
s73060.1	5	15965474	1.070
OAR13_36430806.1	13	33070770	1.069
OAR13_36472387.1	13	33104912	1.056
s36433.1	9	88690261	1.053
s36383.1	5	16420605	1.051
OAR9_82968223.1	9	78287508	1.050
s66343.1	5	15919654	1.032
OAR10_87933952.1	10	80581510	1.027
s46524.1	5	16220530	1.025
OAR15_88007876.1	15	79112023	1.018
s42629.1	13	81009243	1.005
OAR5_19010560.1	5	16550749	1.005
s62871.1	5	16348376	0.998
OAR3_42638679.1	3	39896772	0.993
s56138.1	8	83793039	0.985
s10853.1	5	16080027	0.983
OAR15_87133589.1	15	78508620	0.962
OAR3_117652254.1	3	110467014	0.960
OAR1_197916874.1	1	183420073	0.960
OAR10_86238973.1	10	79062231	0.957
OAR10_90473709.1	10	82888777	0.951
OAR3_117760271.1	3	110513933	0.935
s42422.1	17	61154285	0.932
s49833.1	15	13484995	0.927
OAR15_76452305.1	15	70545475	0.913
OAR14_68402170.1	14	61871888	0.910
OAR17_68457670.1	17	62847208	0.904
OAR13_38704338.1	13	35304376	0.903
s30590.1	1	183676068	0.902
OAR18_59577534.1	18	55762626	0.900
s05690.1	5	16154097	0.898
OAR13_81471049.1	13	75639489	0.896
OAR1_197887718.1	1	183403105	0.894
OAR1_198163788.1	1	183602489	0.893
OAR13_36331465.1	13	32978582	0.890
OAR9_84029059.1	9	79329327	0.890
OAR3_117808530.1	3	110553987	0.886
DU462820_330.1	13	75619811	0.885
OAR15_87767502.1	15	79011094	0.884
OAR15_70943760.1	15	65539039	0.883
OAR1_198260896_X.1	1	183699643	0.875
OAR15_76419512.1	15	70507717	0.875
OAR10_87894196.1	10	80545891	0.875

s38680.1	5	16520048	0.874
OAR13_38680856.1	13	35275288	0.870
OAR3_117626019.1	3	110431071	0.870
s50822.1	15	74721692	0.867
OAR10_90517623.1	10	82933307	0.864
DU464218_590.1	1	183575060	0.859
OAR7_53827294.1	7	48729602	0.857
s62462.1	5	16684125	0.855
s09288.1	1	183640650	0.855
s71442.1	10	82994149	0.855
OAR17_68394746.1	17	62776465	0.854
s53371.1	10	74752408	0.853
OAR17_33260430.1	17	30423316	0.853
OAR3_117858874.1	3	110608833	0.850
s40432.1	14	56451397	0.847
OAR9_94123682.1	9	88593992	0.846
s70525.1	10	79035505	0.843
s51450.1	17	62916211	0.841
s47499.1	10	84137492	0.841
s49282.1	5	16600058	0.832
s58402.1	15	78563409	0.831
OAR13_36256194.1	13	32901567	0.830
OAR22_45509727.1	22	40482146	0.829
s19862.1	15	74618189	0.828
OAR10_82059174.1	10	74893559	0.825
OAR1_245994598.1	1	228281615	0.822
OAR3_118131192.1	3	110878376	0.822
OAR13_38906100.1	13	35507432	0.817
OAR1_198428172.1	1	183846854	0.817
s35627.1	1	183787553	0.815
OAR5_87409839_X.1	5	79542631	0.814
s55347.1	1	183336939	0.813
OAR15_13425521.1	15	13417873	0.810
OAR3_118053045.1	3	110800161	0.810
OAR4_10748026.1	4	10609123	0.809
OAR17_68386597.1	17	62768834	0.809
OAR3_118148991.1	3	110894443	0.809
s56494.1	5	16378679	0.809
s10018.1	1	228225992	0.808
OAR1_245956292_X.1	1	228249737	0.808
OAR1_198322856.1	1	183743001	0.807
OAR3_118084612.1	3	110828511	0.805
OAR1_198090603.1	1	183530032	0.803
OAR23_9974468.1	23	9065236	0.802

OAR1_198449920.1	1	183874319	0.802
s73818.1	14	56425593	0.800
OAR15_87249598.1	15	78626657	0.796
OAR14_27453017.1	14	26280146	0.796
s47327.1	13	55517015	0.796
OAR8_82107007.1	8	76065213	0.795
s54635.1	3	214112448	0.793
s67655.1	15	74641941	0.792
OAR14_27438486.1	14	26265288	0.792
OAR4_80605717.1	4	76007878	0.791
OAR9_94172968.1	9	88641698	0.786
s30457.1	1	183459924	0.786
OAR6_44801570.1	6	40113551	0.785
s04032.1	9	88661518	0.785
s07843.1	9	78510584	0.784
s64674.1	8	76588334	0.783
OAR15_80559047.1	15	74347537	0.782
OAR3_89830043.1	3	84882586	0.781
s00079.1	3	93405961	0.781
OAR10_86176235.1	10	78950748	0.780
OAR15_81031438.1	15	74735737	0.777
OAR3_117843556.1	3	110589829	0.777
OAR14_27342805.1	14	26174255	0.777
OAR14_27356556.1	14	26186832	0.777
OAR13_87444196.1	13	81364321	0.774
s00941.1	15	74636302	0.773
OAR7_47161420.1	7	42551974	0.772
s74549.1	19	29047903	0.771
OAR2_61008309.1	2	56714900	0.771
OAR4_10761398_X.1	4	10623464	0.770
s66058.1	9	32100676	0.769
OAR3_89811036.1	3	84864209	0.768
OAR10_12172332.1	10	13605338	0.768
OAR15_80591359.1	15	74380848	0.768
OAR5_87470215.1	5	79603510	0.766
s20638.1	5	16618176	0.764
OAR18_62455560.1	18	58445827	0.762
OAR8_81217085.1	8	75305198	0.762
s49955.1	3	93554720	0.762
OAR8_90425834.1	8	83842627	0.762
OAR4_91329005.1	4	85930010	0.761
OAR13_81364186.1	13	75530926	0.760
OAR13_87230941.1	13	81137021	0.760
s25024.1	17	61092263	0.759

s56763.1	5	15912332	0.759
OAR15_80994401.1	15	74700420	0.759
OAR9_82702203.1	9	77991830	0.758
OAR9_82953999.1	9	78269811	0.757
OAR9_83994635.1	9	79292813	0.757
OAR14_27463865.1	14	26293894	0.757
OAR10_12146157.1	10	13579925	0.756
s16333.1	13	66077976	0.756
OAR3_127956387_X.1	3	120178240	0.756
OAR9_6883350.1	9	7021673	0.756
OAR16_33435828.1	16	30720540	0.755
OAR6_64832803.1	6	58850066	0.755
s55067.1	3	213928939	0.755
OAR15_76595841.1	15	70691131	0.754
OAR3_98710538.1	3	93004742	0.752
OAR3_117907362.1	3	110649847	0.751
OAR20_50695807.1	20	46597682	0.750
OAR15_70998725.1	15	65593216	0.748
OAR8_22222478.1	8	19757414	0.747
OAR9_82428531.1	9	77756891	0.745
s67952.1	3	93522006	0.743
OAR2_60972128.1	2	56679411	0.740
OAR13_39005670.1	13	35610109	0.738
s27778.1	10	76069138	0.738
s67744.1	10	84100127	0.738
OAR3_117968696.1	3	110722632	0.737
DU231119_249.1	9	7004850	0.737
OAR1_245881272.1	1	228182650	0.735
OAR16_42321024.1	16	38952447	0.735
OAR8_22391216.1	8	19808188	0.735
OAR8_84976102.1	8	78812357	0.733
OAR3_127924329_X.1	3	120136049	0.732
OAR13_87268558.1	13	81176490	0.731
OAR9_94310472.1	9	88775547	0.731
OAR8_58028748.1	8	54142002	0.730
OAR7_53759993.1	7	48695180	0.730
OAR5_64796936.1	5	58959691	0.730
OAR9_82309015.1	9	77648954	0.729
OAR6_64965984.1	6	58974752	0.729
OAR15_78509799.1	15	72430785	0.728
OAR9_82293368.1	9	77630787	0.726
OAR3_117945750.1	3	110692574	0.726
OAR14_68366733.1	14	61840283	0.725
s38745.1	3	214081723	0.724

OAR9_82282148_X.1	9	77619742	0.723
OAR7_53909085.1	7	48811214	0.720
OAR2_65726543.1	2	61275543	0.720
OAR18_59593056.1	18	55777542	0.719
s21089.1	17	60192558	0.718
OAR17_68362876.1	17	62746209	0.717
OAR7_53952286.1	7	48854441	0.717
OAR10_86298190.1	10	79129227	0.716
OAR10_84250273.1	10	77042069	0.715
OAR14_27408870.1	14	26238454	0.715
OAR12_84459237.1	12	76720048	0.715
s18674.1	13	35248112	0.714
OAR3_117600112.1	3	110405090	0.714
OAR4_37225390.1	4	35004679	0.714
OAR10_57242083.1	10	56100245	0.712
OAR10_84310189.1	10	77101531	0.712
s62974.1	12	69228088	0.712
s17550.1	7	42463209	0.711
OAR3_83971441.1	3	79424414	0.711
s45715.1	5	58992351	0.710
s70599.1	15	73730180	0.710
OAR10_91895029.1	10	84261836	0.709
s01733.1	19	29068797	0.709
OAR15_13465084.1	15	13456857	0.708
s15797.1	3	110770412	0.707
OAR15_76379434.1	15	70452674	0.705
s46185.1	13	78420224	0.705
s38430.1	12	75200203	0.703
s16840.1	5	59000838	0.703
OAR8_51076568.1	8	47596948	0.703
OAR4_25295648.1	4	24132337	0.702
OAR4_11378770.1	4	11236528	0.701
s40911.1	6	24065226	0.701
s62708.1	12	76777020	0.700
OAR15_13340852.1	15	13332149	0.700
OAR8_81299856.1	8	75379150	0.699
OAR8_22199299_X.1	8	19734405	0.699
s06150.1	2	244899259	0.699
OAR14_68472862.1	14	61901925	0.697
OAR9_1718056.1	9	2004658	0.697
OAR15_13536503.1	15	13528595	0.697
OAR13_87564322.1	13	81422743	0.697
OAR1_198462100.1	1	183887136	0.697
OAR9_6943231.1	9	7080616	0.696

s13821.1	5	59203793	0.694
s19512.1	3	76037302	0.694
OAR17_68309863.1	17	62688843	0.694
OAR13_38886449.1	13	35490974	0.694
OAR3_82409790.1	3	77879156	0.693
s49508.1	18	58463660	0.693
OAR3_118209937.1	3	110955269	0.693
OAR3_232015599.1	3	214046599	0.691
OAR11_32450209.1	11	30567032	0.691
OAR8_84987023.1	8	78814972	0.690
OAR3_43953410.1	3	41095357	0.690
OAR25_19253472_X.1	25	18533982	0.690
s22433.1	9	82762868	0.689
s42597.1	3	93819638	0.689
OAR14_68319489.1	14	61794245	0.688
s67424.1	3	84822891	0.688
OAR4_73786063.1	4	69811362	0.688
OAR1_198064156.1	1	183513051	0.687
s01719.1	7	48685941	0.687
s62083.1	13	36011422	0.686
s38320.1	8	51842542	0.686
OAR3_79159004.1	3	74929284	0.684
s75876.1	21	42601748	0.684
s70532.1	5	58917824	0.683
OAR9_33735501_X.1	9	32212468	0.682
s02448.1	22	40450104	0.682
s34861.1	5	79693742	0.681
OAR21_46682445.1	21	42300786	0.681
s03078.1	13	33058560	0.681
OAR18_59519283.1	18	55698328	0.680
OAR7_18503023.1	7	17777251	0.679
OAR3_190383205.1	3	177408460	0.678
s35258.1	3	217238883	0.677
s14938.1	9	795580	0.677
s72653.1	15	31723078	0.676
OAR3_79206128.1	3	74982457	0.676
OAR9_33777151.1	9	32258653	0.676
OAR8_60234503.1	8	56216655	0.676
OAR15_33211632.1	15	31683921	0.676
s42116.1	13	81454801	0.676
OAR12_84767663.1	12	76845918	0.675
s14087.1	3	93271832	0.674
OAR6_65100822.1	6	59116808	0.674
s57260.1	6	40223479	0.674

OAR21_34333027.1	21	30751827	0.674
OAR11_18625918.1	11	18255835	0.673
OAR7_53985984.1	7	48887292	0.673
OAR13_36318213.1	13	32965686	0.672
s00653.1	1	231652278	0.672
OAR9_82178363.1	9	77571488	0.672
OAR19_33593848.1	19	31915771	0.671
s61765.1	18	60674746	0.670
OAR12_42565904.1	12	38201807	0.670
s50307.1	24	7721963	0.668
OAR4_11392839.1	4	11250213	0.667
OAR1_107287431.1	1	99972078	0.667
OAR4_93067608.1	4	87649810	0.667
s34694.1	2	244814221	0.666
OAR17_33279012.1	17	30441923	0.666
OAR3_84033269.1	3	79473981	0.666
s03253.1	24	7615311	0.665
s43578.1	3	213806251	0.664
OAR4_80559387.1	4	75968047	0.663
OAR8_84946107.1	8	78766776	0.663
OAR15_33293466_X.1	15	31763433	0.663
OAR16_27229721.1	16	25021431	0.662
s02743.1	14	56495857	0.662
OAR9_1734865.1	9	1988218	0.661
OAR8_91701722.1	8	85040138	0.660
OAR21_46936641.1	21	42442018	0.659
s07697.1	12	38391949	0.659
OAR9_82171170.1	9	77563420	0.658
OAR2_141478977_X.1	2	132967710	0.658
OAR4_83374739.1	4	78627494	0.658
OAR1_206552578.1	1	191356092	0.658
OAR12_67663336.1	12	61208417	0.657
OAR3_84111819.1	3	79547932	0.657
OAR3_99622529.1	3	93811641	0.655
OAR3_82349536.1	3	77817377	0.655
OAR3_231922518.1	3	213955526	0.654
OAR4_80633497.1	4	76037014	0.654
s64982.1	3	93725974	0.654
s09178.1	3	93330223	0.653
OAR8_56047895.1	8	52121715	0.652
OAR19_33417302.1	19	31737627	0.651
OAR3_84073899.1	3	79511180	0.651
OAR15_22404265.1	15	21482600	0.650
OAR3_152693749.1	3	142848742	0.650

s66575.1	4	47439380	0.650
OAR6_65237136.1	6	59255622	0.649
s08413.1	3	214006806	0.649
OAR3_225655674.1	3	207999099	0.648
OAR5_104164895.1	5	95711861	0.647
s20190.1	2	244962782	0.647
OAR8_55825703.1	8	51981196	0.646
s26777.1	3	213906966	0.645
s37379.1	21	30761604	0.644
OAR15_78019848.1	15	71981656	0.643
s66828.1	15	65522727	0.642
s25926.1	13	81223444	0.642
OAR8_93512767.1	8	86728596	0.641
s32677.1	19	31833784	0.641
s28437.1	17	62982754	0.641
OAR1_81160492.1	1	75981581	0.640
OAR8_91759596.1	8	85080887	0.640
OAR15_32816326.1	15	31325582	0.640
OAR13_87648967.1	13	81506987	0.639
s25636.1	17	61218530	0.639
s33447.1	7	17324345	0.639
s71801.1	13	35418640	0.639
s14434.1	13	77724530	0.638
OAR1_206588453.1	1	191393205	0.638
OAR9_97890623.1	9	91993061	0.637
OAR4_10829975.1	4	10685933	0.637
s10703.1	21	42434301	0.637
OAR4_83356798.1	4	78611633	0.637
s28673.1	8	80327331	0.637
s26313.1	3	79415127	0.637
OAR8_22272619.1	8	19679596	0.635
OAR2_180722401.1	2	170471855	0.634
OAR8_93540310.1	8	86753491	0.634
OAR15_77948911.1	15	71913377	0.634
OAR1_249572898.1	1	231602195	0.634
OAR9_96141857.1	9	90491974	0.634
OAR2_172701586.1	2	163070840	0.634
s01640.1	2	135178922	0.632
OAR8_82139676.1	8	76094721	0.632
s50043.1	3	217268499	0.632
OAR12_72924306.1	12	66252130	0.632
s05273.1	16	39057236	0.631
OAR13_81410207.1	13	75577565	0.631
OAR18_64788162_X.1	18	60788242	0.631

DU310703_497.1	12	75121332	0.631
OAR10_28149069.1	10	28136160	0.631
OAR9_8935577.1	9	9072827	0.630
OAR9_6774170.1	9	6922921	0.630
OAR8_93573717.1	8	86788358	0.629
s43870.1	2	132934057	0.629
OAR17_31561091.1	17	28790576	0.628
OAR19_14489436.1	19	13918425	0.628
OAR2_259002206.1	2	245049692	0.628
s20108.1	17	65695441	0.628
OAR3_190368631.1	3	177394170	0.627
s70619.1	16	9109431	0.626
OAR15_78548290.1	15	72470215	0.626
OAR19_46690670.1	19	44350807	0.625
OAR3_152741614.1	3	142894678	0.625
OAR1_145252178.1	1	134152602	0.625
OAR2_180971216.1	2	170637499	0.625
OAR2_13925483.1	2	14508559	0.625
OAR3_138331159.1	3	129685397	0.624
s11510.1	9	39402216	0.624
OAR25_19329042.1	25	18624514	0.624
OAR4_26645098.1	4	25358759	0.623
OAR1_245847989.1	1	228160493	0.622
OAR6_76407642.1	6	69901252	0.622
s67834.1	21	42502482	0.622
OAR12_42693133.1	12	38285702	0.622
OAR12_42864343.1	12	38461703	0.622
OAR1_245769021.1	1	228089416	0.621
OAR1_245789135.1	1	228106358	0.621
OAR2_165305793.1	2	155889712	0.621
s57541.1	10	13560775	0.621
s13618.1	2	132868782	0.621
OAR9_33070898.1	9	31552301	0.621
s53056.1	2	40839676	0.620
OAR12_76153608.1	12	69326479	0.619
s09462.1	3	104064729	0.619
OAR13_70737254.1	13	65652628	0.619
s18401.1	13	65660964	0.619
OAR8_55643574.1	8	51778519	0.619
OAR6_64875580_X.1	6	58892237	0.618
OAR3_195236801.1	3	181251741	0.618
s39564.1	13	43797381	0.618
s36428.1	3	25612070	0.617
OAR19_33355170.1	19	31674797	0.617

OAR9_8924567.1	9	9058127	0.616
s19461.1	22	39739539	0.616
OAR3_150667114.1	3	140853334	0.616
OAR8_22309726.1	8	19715836	0.615
s59697.1	13	77685416	0.615
OAR16_42448158.1	16	39077896	0.615
s15337.1	3	143068596	0.614
OAR7_18402440.1	7	17681312	0.614
OAR12_42841466.1	12	38432263	0.614
s40856.1	11	41441937	0.613
OAR13_60486404.1	13	55508708	0.613
OAR3_195088663.1	3	181113434	0.613
OAR7_47189570.1	7	42580713	0.612
OAR1_268122939.1	1	248275678	0.612
OAR9_6934929.1	9	7073202	0.612
s35677.1	15	13579528	0.612
OAR2_13939688.1	2	14523912	0.612
OAR2_63283575.1	2	58905801	0.611
OAR4_93263585.1	4	87830835	0.611
OAR8_86488410.1	8	80247025	0.611
s33957.1	11	41563065	0.611
OAR3_80356382_X.1	3	76052424	0.610
s06109.1	15	74597812	0.609
OAR10_87814152.1	10	80465758	0.609
s16659.1	13	81067937	0.609
s10362.1	19	31777619	0.608
s26719.1	12	69269910	0.607
s13370.1	1	108116540	0.607
s21640.1	25	13152201	0.607
OAR4_73842776_X.1	4	69869475	0.607
OAR10_87867275.1	10	80519356	0.606
s72510.1	8	10725738	0.605
s03117.1	13	77857774	0.605
s36316.1	2	244165892	0.605
OAR8_83343787.1	8	77245074	0.605
s12526.1	13	65479991	0.604
OAR13_39447377.1	13	36045045	0.603
OAR7_53438825.1	7	48378412	0.603
s12156.1	5	59160205	0.603
OAR8_89520460.1	8	83001737	0.603
OAR10_50091915.1	10	49246348	0.602
OAR2_42709839.1	2	40875507	0.602
OAR3_190359838.1	3	177385320	0.602
OAR9_8579331.1	9	8820234	0.602

OAR3_125588829.1	3	117721356	0.602
OAR2_141413285.1	2	132904756	0.601
OAR8_12060950.1	8	10761579	0.600
OAR10_82097240_X.1	10	74924462	0.600
OAR1_116830364.1	1	108001622	0.600
OAR15_78164304.1	15	72091092	0.600
OAR4_36981043.1	4	34937344	0.600
DU398082_567.1	7	48962917	0.600
s27142.1	16	66542249	0.599
OAR18_20398726.1	18	19898256	0.599
OAR4_93024080.1	4	87602738	0.599
OAR22_10482614.1	22	8739841	0.599
OAR22_34323192.1	22	29843623	0.599
OAR10_28087786.1	10	28071590	0.598
OAR16_33478147.1	16	30847022	0.598
OAR26_9403466.1	26	7276882	0.598
s11660.1	11	41465606	0.597
s10816.1	13	68732696	0.597
OAR7_47322813.1	7	42706326	0.597
OAR10_29381795.1	10	29344224	0.597
s02782.1	12	69314979	0.597
OAR8_56100402.1	8	52180896	0.597
OAR16_43190112.1	16	39708223	0.597
s01629.1	1	2721556	0.595
s20408.1	3	213849396	0.595
OAR2_60563723.1	2	56274688	0.594
s54502.1	11	12686482	0.594
s61162.1	22	40552713	0.593
OAR8_57964281.1	8	54076612	0.593
OAR4_87332839.1	4	82212446	0.593
OAR19_60184770.1	19	56634864	0.593
OAR5_96790272.1	5	88746609	0.593
OAR8_67149281.1	8	62355310	0.592
OAR10_40627671.1	10	39816919	0.592
OAR2_144002704.1	2	135391568	0.592
OAR1_156816942.1	1	145279459	0.591
OAR2_43037062.1	2	41162381	0.591
OAR17_33357382.1	17	30468599	0.591
OAR7_18423084.1	7	17701363	0.591
OAR18_59003703.1	18	55210183	0.591
s74680.1	18	59200949	0.590
s53519.1	18	29717895	0.590
s16714.1	2	163140231	0.590
OAR3_84348827.1	3	79772321	0.590

OAR19_33525291.1	19	31850022	0.589
OAR5_36644948.1	5	33090257	0.589
OAR2_65914681.1	2	61456748	0.589
OAR22_29183207.1	22	24858581	0.588
OAR8_11551388.1	8	10368179	0.588
OAR6_27400136.1	6	24006078	0.587
OAR16_37539041.1	16	34550197	0.587
s64995.1	8	75417603	0.586
s49373.1	20	46556750	0.586
OAR2_95050543.1	2	89189237	0.586
OAR6_27332730.1	6	23939264	0.585
OAR1_152473283.1	1	141297942	0.585
OAR3_110555269_X.1	3	104173689	0.585
OAR16_44102697.1	16	40583350	0.585
OAR10_29341212.1	10	29304176	0.584
s42960.1	3	213824504	0.584
OAR4_80509083.1	4	75911464	0.583
OAR10_49684438.1	10	48900679	0.583
OAR3_84148810.1	3	79584309	0.583
OAR17_67071168.1	17	61513077	0.583
OAR11_12480303.1	11	12728167	0.583
s11336.1	4	87572495	0.583
OAR12_84842455.1	12	76751672	0.583
OAR23_9959034.1	23	9048593	0.583
OAR21_13135613.1	21	11577229	0.582
OAR23_42222982.1	23	39828270	0.582
OAR2_252085914.1	2	238675170	0.582
OAR8_58134022.1	8	54236077	0.581
s29911.1	18	10929367	0.581
s05828.1	6	12467500	0.581
OAR21_34358292.1	21	30779239	0.581
OAR9_41429299.1	9	39453432	0.581
OAR15_33315132.1	15	31784781	0.580
OAR15_13214168.1	15	13200152	0.580
OAR16_33588226.1	16	30948356	0.580
OAR3_195114301.1	3	181139296	0.580
OAR3_44188902.1	3	41281473	0.580
OAR3_118335841.1	3	111119165	0.579
OAR8_56107289.1	8	52190725	0.579
OAR16_33317775.1	16	30655866	0.579
OAR3_196830362.1	3	182820486	0.579
OAR6_65117122.1	6	59133189	0.578
s35395.1	15	13615819	0.578
OAR18_11985596.1	18	12026856	0.578

OAR2_34260595.1	2	32949467	0.578
s49110.1	18	58474543	0.577
OAR1_52460849.1	1	50316433	0.577
s05435.1	18	60735654	0.577
OAR5_104273659.1	5	95731940	0.577
OAR24_42942982.1	24	39726323	0.577
s41580.1	2	32913281	0.577
s44712.1	2	135753568	0.577
OAR15_8329424.1	15	8616919	0.576
OAR7_53702492.1	7	48639590	0.576
OAR17_31784331.1	17	29015377	0.576
OAR16_44166560.1	16	40650762	0.575
OAR5_36627156.1	5	33071203	0.575
OAR8_83390411.1	8	77284736	0.575
s73962.1	3	93903353	0.575
OAR15_76652734.1	15	70742236	0.574
OAR6_55423607.1	6	50127083	0.574
OAR12_42582975.1	12	38218663	0.574
s14577.1	7	26755264	0.574
s61354.1	5	90586440	0.574
s58484.1	2	165546958	0.574
OAR17_65728611.1	17	60199858	0.574
OAR8_55597205.1	8	51730681	0.573
OAR16_42499047.1	16	39127633	0.573
OAR1_181982373.1	1	168760749	0.573
OAR1_117069007.1	1	108272813	0.573
s74362.1	3	111011549	0.573
s10664.1	2	244129054	0.573
s40989.1	24	33172658	0.573
OAR13_70672871.1	13	65588250	0.573
s03142.1	3	217226491	0.573
s56877.1	17	65662344	0.572
OAR3_78052239_X.1	3	73892462	0.572
OAR3_128319473.1	3	120414509	0.572
OAR1_241598383.1	1	224047352	0.572
s70623.1	10	24789023	0.572
OAR2_166515103.1	2	157090967	0.572
OAR8_86639423.1	8	80390056	0.572
OAR3_128369653.1	3	120461206	0.572
OAR15_53721488.1	15	49215124	0.572
OAR4_26579960.1	4	25292594	0.572
s54026.1	3	151972015	0.571
s07047.1	20	46575517	0.571
OAR3_195631696.1	3	181650708	0.571

OAR13_87428154.1	13	81349148	0.571
s65742.1	3	208052819	0.571
s17708.1	1	53355068	0.570
OAR2_68196664.1	2	63720116	0.570
OAR10_29448537.1	10	29415140	0.570
OAR3_16758206.1	3	15525958	0.570
OAR12_75251826.1	12	68481603	0.570
OAR26_40820405.1	26	35876688	0.570
OAR5_94270780.1	5	86055067	0.570
OAR2_146760496.1	2	138159566	0.569
OAR12_63367607.1	12	56957221	0.569
OAR11_3180902.1	11	3834100	0.569
OAR9_9093388.1	9	9264542	0.569
OAR3_89640427.1	3	84689397	0.569
s20727.1	16	40527757	0.569
s60404.1	2	245007283	0.569
OAR7_53659419_X.1	7	48597074	0.568
OAR5_80840861.1	5	73586339	0.568
s19664.1	9	79378534	0.568
OAR26_41000588.1	26	36074023	0.568
DU345394_399.1	18	60904691	0.568
OAR2_23428619.1	2	23090794	0.568
OAR15_28647683.1	15	27335675	0.568
s46664.1	3	217310965	0.567
s65675.1	8	54187572	0.567
OAR15_32800538.1	15	31309312	0.567
s19317.1	2	238602466	0.567
s67284.1	25	40426700	0.566
OAR2_142416977.1	2	133928107	0.566
s21764.1	1	107955452	0.566
OAR3_87830734.1	3	82884212	0.566
OAR5_23712613.1	5	21070914	0.566
OAR1_124203566.1	1	115232883	0.566
s57800.1	11	47905026	0.566
s28575.1	21	42551316	0.565
OAR9_14653377.1	9	14303907	0.565
OAR4_94588384.1	4	89090017	0.565
s24365.1	5	36459832	0.565
s39959.1	2	244947713	0.565
OAR6_43010674.1	6	38617412	0.565
OAR17_25389294.1	17	22988961	0.565
OAR16_33405502.1	16	30692759	0.565
s67702.1	13	66132902	0.565
s69776.1	2	31564277	0.565

OAR1_52636268.1	1	50494736	0.564
OAR3_197060241.1	3	183051361	0.564
OAR2_61130563.1	2	56840997	0.564
s10098.1	13	77634996	0.564
OAR18_20569924.1	18	20071226	0.564
OAR2_48093430.1	2	45357951	0.564
s04347.1	1	42856505	0.564
OAR3_196681113.1	3	182669294	0.564
OAR3_122207054.1	3	114648138	0.563
OAR4_10699994.1	4	10559577	0.563
OAR12_42742443_X.1	12	38336928	0.563
s49449.1	8	19896747	0.563
OAR9_6641948.1	9	6721696	0.563
OAR16_31845248.1	16	29304504	0.563
OAR6_26089154.1	6	22814552	0.562
OAR5_93445511_X.1	5	85245598	0.562
OAR15_79929526.1	15	73750680	0.562
s20320.1	2	245030239	0.562
OAR19_22142283.1	19	28809608	0.562
OAR3_99006940.1	3	93277829	0.561
OAR2_180932811.1	2	170602376	0.561
OAR17_44358460.1	17	41064820	0.561
s56460.1	10	27645248	0.561
OAR7_50099626.1	7	45368868	0.561
s45695.1	3	4648352	0.561
OAR1_145163431.1	1	134137436	0.560
OAR8_62203494_X.1	8	57876220	0.560
OAR4_94538015.1	4	89040955	0.560
s29286.1	4	117266371	0.560
OAR12_28635024.1	12	25171224	0.560
OAR1_107239370.1	1	99931717	0.559
OAR4_10443301.1	4	10281638	0.559
OAR10_86330122.1	10	79161469	0.559
OAR4_83328517.1	4	78581178	0.559
s01421.1	3	177456644	0.559
OAR4_52735300.1	4	49919893	0.558
OAR8_11646058.1	8	10461730	0.558
OAR15_70840858.1	15	65425993	0.558
OAR8_83502542.1	8	77384602	0.558
OAR3_196904777.1	3	182900674	0.557
OAR3_196913312.1	3	182916410	0.557
s67158.1	25	7727709	0.557
s27686.1	7	49009028	0.557
OAR1_206496404.1	1	191302900	0.556

s29150.1	4	117204244	0.556
OAR2_91693967.1	2	86217927	0.555
OAR9_96060950.1	9	90516874	0.555
OAR18_64851953.1	18	60853888	0.555
OAR1_52518679.1	1	50373341	0.555
OAR6_42945420.1	6	38567335	0.555
OAR14_57922732.1	6	38580198	0.555
s02472.1	20	15603841	0.554
OAR2_165282874.1	2	155866494	0.554
OAR14_39759149.1	14	38170832	0.554
OAR2_118498709.1	2	110291246	0.554
OAR22_10514814.1	22	8774021	0.553
OAR3_84154568.1	3	79592460	0.553
OAR1_117144883.1	1	108334841	0.553
OAR8_55769107.1	8	51921694	0.553
OAR1_52744886.1	1	50596811	0.552
s66528.1	20	46649856	0.552
s25789.1	1	50306410	0.552
OAR21_48060059.1	21	43472640	0.551
s25030.1	9	31534631	0.551
s52346.1	12	61401931	0.551
OAR3_127882408.1	3	120095387	0.551
OAR13_60423450.1	13	55558769	0.551
s10695.1	15	27385126	0.551
OAR10_24807423.1	10	24900528	0.551
s45296.1	10	19465704	0.551
s30200.1	17	62654616	0.551
OAR6_43104220.1	6	38706958	0.551
s58187.1	22	16538861	0.550
OAR19_48025918.1	19	45596524	0.550
OAR2_245949843.1	2	232863244	0.550
s12587.1	3	93343121	0.550
OAR9_89266462.1	9	84373507	0.550
OAR13_73857254.1	13	68662488	0.549
s12995.1	24	7667607	0.549
OAR3_96268784.1	3	90654749	0.549
s34702.1	20	46681742	0.549
OAR1_246159875.1	1	228372242	0.549
OAR1_52559106.1	1	50412664	0.549
OAR3_125694577.1	3	117818595	0.549
OAR6_15051305.1	6	12499974	0.549
OAR1_214424851.1	1	198653992	0.548
OAR2_43139454.1	2	41262244	0.548
OAR8_22174396.1	8	19674317	0.548

OAR11_18815864.1	11	18433474	0.548
OAR8_27416478.1	8	24996355	0.548
s13514.1	9	32128668	0.548
OAR2_147566610.1	2	138966957	0.548
OAR8_57948794.1	8	54058725	0.547
s49062.1	18	46100361	0.547
s27865.1	22	43220669	0.547
OAR18_32289792.1	18	30930994	0.547
OAR9_8904760.1	9	9039083	0.547
OAR10_30205238.1	10	30157078	0.547
OAR16_10200326.1	16	9251385	0.547
s54313.1	2	13836112	0.547
OAR6_64921019.1	6	58934336	0.547
OAR25_19360132.1	25	18598726	0.546
OAR17_64889221.1	17	59447245	0.546
OAR4_107786618.1	4	101313300	0.546
s23170.1	11	47799943	0.546
s62006.1	3	4401530	0.546
s71169.1	3	129759745	0.546
s16589.1	7	20691591	0.546
s59565.1	16	30667706	0.545
OAR14_68274557.1	14	61754095	0.545
OAR16_44226044.1	16	40705136	0.545
OAR2_145195113.1	2	136512593	0.545
s51045.1	3	25632751	0.545
OAR1_145170621.1	1	134127517	0.545
s58543.1	13	12265286	0.545
OAR8_12151702.1	8	10853693	0.545
OAR9_8876882.1	9	9011390	0.544
OAR3_95822218.1	3	90220353	0.544
s44167.1	2	243153063	0.544
OAR4_96453312.1	4	90884717	0.544
OAR5_30583112.1	5	27557782	0.544
OAR16_33224073.1	16	30559452	0.544
s51915.1	19	44131540	0.543
OAR15_76711224.1	15	70799094	0.543
OAR5_40288441.1	5	36543472	0.543
s69481.1	2	14496716	0.543
OAR1_30798950.1	1	30143388	0.543
OAR22_10669315.1	22	8916969	0.543
OAR1_246064756.1	1	228358337	0.543
OAR1_43042460.1	1	41564355	0.543
OAR2_60936578.1	2	56639600	0.543
OAR5_87240867.1	5	79373938	0.543

OAR2_160737599.1	2	151588637	0.543
OAR4_91373356.1	4	85975743	0.542
OAR7_53275088.1	7	48234767	0.542
s70054.1	16	9207808	0.542
s05264.1	13	65420796	0.542
s45070.1	7	62331047	0.542
OAR1_42968648.1	1	41492360	0.542
OAR1_214417389.1	1	198646310	0.542
OAR6_55532476.1	6	50226730	0.542
OAR3_15518578.1	3	14371537	0.542
OAR16_44015351.1	16	40503694	0.541
OAR12_42512998.1	12	38146891	0.541
OAR2_63274739.1	2	58896602	0.541
OAR16_28163128.1	16	25940847	0.541
OAR1_249629551.1	1	231659828	0.541
OAR21_34401682.1	21	30822358	0.541
OAR1_117122442.1	1	108321166	0.541
OAR15_80616593.1	15	74407015	0.541
s07317.1	14	54848584	0.540
OAR16_43133409.1	16	39659615	0.540
OAR3_195291305.1	3	181380872	0.540
OAR10_29389966_X.1	10	29353089	0.540
OAR26_21025118.1	26	17824781	0.540
s62730.1	5	59295706	0.540
OAR16_42159705.1	16	38851083	0.539
OAR7_21639936.1	7	20784154	0.539
s14191.1	9	32071504	0.539
OAR2_140167981.1	2	131761141	0.539
s61003.1	3	154765061	0.538
s62032.1	2	170563260	0.538
OAR3_44233744.1	3	41320283	0.538
OAR16_37467394.1	16	34475296	0.538
OAR11_50751433.1	11	47776663	0.538
OAR15_78598295.1	15	72520678	0.538
s24065.1	4	103237931	0.538
OAR3_78893934.1	3	74725586	0.538
OAR1_79775846.1	1	74542343	0.538
OAR2_180650246.1	2	170389751	0.537
s09340.1	15	74546560	0.537
s39945.1	7	44271809	0.537
OAR4_80646791.1	4	76055323	0.537
OAR3_196880003.1	3	182867529	0.536
OAR6_76377079.1	6	69873262	0.536
OAR4_80416194.1	4	75818223	0.536

s16511.1	3	181316897	0.536
OAR17_35455576.1	17	32500054	0.536
s18835.1	23	2697393	0.536
s43545.1	15	74499985	0.536
s27604.1	1	198629915	0.536
OAR9_41445839.1	9	39471650	0.536
OAR8_27446520.1	8	25025767	0.535
OAR5_55127059.1	5	50841057	0.535
OAR2_180261787.1	2	170218116	0.535
OAR2_180267656.1	2	170221187	0.535
OAR3_152090860.1	3	142262633	0.535
OAR3_4471127.1	3	4483359	0.535
OAR22_32799694.1	22	28489864	0.535
OAR6_44719458.1	6	40035197	0.535
OAR10_48888461.1	10	48124132	0.535
s06058.1	4	39035709	0.534
OAR16_15575456.1	16	14331214	0.534
s58020.1	3	41397228	0.534
OAR4_10789078.1	4	10644494	0.534
OAR3_87788635.1	3	82845251	0.534
OAR11_18823250.1	11	18440783	0.534
OAR8_96563089.1	8	89573903	0.534
OAR13_81329564.1	13	75498647	0.533
OAR12_43172807.1	12	38765389	0.533
OAR3_118302402.1	3	111053851	0.533
s61032.1	18	29723883	0.533
OAR3_195161577.1	3	181180472	0.533
OAR5_40108847.1	5	36361620	0.532
OAR6_67376317.1	6	61138606	0.532
OAR3_156734590.1	3	146887886	0.532
OAR5_94259136.1	5	86043483	0.532
OAR1_214229899.1	1	198470222	0.531
OAR15_38872932.1	15	37026092	0.531
OAR10_50107347.1	10	49263376	0.531
OAR26_35965231.1	26	31624872	0.531
OAR3_156691225.1	3	146838020	0.531
s33445.1	24	25240220	0.531
OAR6_44473865.1	6	39789613	0.531
OAR6_23189054.1	6	20222258	0.531
OAR3_4694801.1	3	4700819	0.530
OAR3_197179842.1	3	183150221	0.530
OAR2_165382716.1	2	155969590	0.530
OAR3_195197327.1	3	181219459	0.530
s34288.1	1	191440155	0.530

OAR15_20586574.1	15	19913859	0.529
OAR3_225570777.1	3	207988763	0.529
OAR6_80988051.1	6	74188622	0.529
s14772.1	3	5146200	0.529
OAR19_33531772.1	19	31856390	0.529
s26710.1	19	53596723	0.529
OAR3_156766007_X.1	3	146921476	0.529
OAR2_259153647.1	2	245206162	0.529
s53951.1	6	23688531	0.528
OAR23_38365072.1	23	36293219	0.528
OAR12_43199598.1	12	38805757	0.528
OAR10_48898826.1	10	48135136	0.528
OAR18_68792365.1	18	64909980	0.528
s69625.1	14	23274589	0.528
OAR12_62571692.1	12	56214960	0.528
OAR3_44291290.1	3	41375055	0.527
OAR4_27853334.1	4	26449971	0.527
OAR18_34149127.1	18	32659588	0.527
s71544.1	17	60954982	0.527
s39351.1	3	183075730	0.527
OAR9_76802154.1	9	72375489	0.526
s19722.1	15	27287086	0.526
OAR6_124660937.1	6	109711650	0.526
s23582.1	8	28025413	0.526
s08060.1	21	24530208	0.526
OAR9_99774199.1	9	93761494	0.526
OAR3_232134900.1	3	214158027	0.526
OAR7_18356443.1	7	17660376	0.526
OAR10_86141363.1	10	78916131	0.525
s39725.1	10	13487310	0.525
OAR17_29119468.1	8	82471334	0.525
s67277.1	18	59152360	0.525
s04965.1	3	208078768	0.525
OAR13_38561999.1	13	35163552	0.525
s33255.1	4	65021659	0.524
s45053.1	16	70559566	0.524
OAR6_44769116.1	6	40084437	0.524
OAR15_14742464.1	15	14639505	0.524
OAR11_6228500.1	11	6725442	0.524
OAR5_59436109.1	5	54625756	0.523
OAR26_46921474.1	26	41364432	0.523
OAR6_120486822_X.1	6	106042672	0.522
OAR8_88991389.1	8	82529068	0.522
OAR3_95711572.1	3	90106223	0.522

s57574.1	14	56384913	0.522
OAR4_91397094.1	4	85999313	0.522
s29948.1	6	45352624	0.522
s43247.1	1	76041981	0.522
OAR2_131889391.1	2	123671526	0.522
s09832.1	7	48944499	0.521
OAR6_120480090.1	6	106035342	0.521
s31978.1	3	5123441	0.521
s66349.1	1	68199001	0.521
s39735.1	14	19615508	0.520
OAR6_27354451.1	6	23965040	0.520
OAR22_10602337.1	22	8859266	0.519
s75037.1	5	67865996	0.519
s03682.1	12	27035129	0.519
OAR23_32486938.1	23	30809730	0.519
s31825.1	13	35979114	0.519
s45473.1	17	60141056	0.519
s53085.1	3	4543145	0.519
OAR1_281749581.1	1	260357828	0.519
s20188.1	18	29519813	0.519
OAR14_27546419.1	14	26371515	0.518
s21579.1	7	11778527	0.518
OAR3_92910516.1	3	87752928	0.518
s57805.1	26	7244869	0.518
OAR1_181917984.1	1	168696731	0.518
OAR6_76321832.1	6	69827951	0.518
OAR3_53626593.1	3	50929192	0.518
OAR3_122241058.1	3	114684255	0.518
s41600.1	8	28018925	0.518
OAR15_72182875.1	15	66675925	0.518
OAR4_93149584.1	4	87732399	0.518
OAR15_55438774.1	15	50730627	0.518
s73153.1	8	80104991	0.517
OAR10_48546137.1	10	47797098	0.517
s38018.1	13	43846221	0.517
OAR23_3024132.1	23	2762841	0.517
OAR16_33555314.1	16	30922730	0.516
s49903.1	5	59260702	0.516
OAR5_94989910.1	5	86891042	0.516
OAR9_1625333.1	9	1906814	0.516
OAR6_44450940.1	6	39767543	0.516
OAR8_64247515.1	8	59597770	0.515
OAR9_87534142.1	9	82734427	0.515
s40766.1	7	17329557	0.515

OAR16_33184393.1	16	30522362	0.515
OAR2_61094453.1	2	56804497	0.515
OAR5_96574870.1	5	88548058	0.515
s41370.1	3	5062211	0.515
OAR3_57525359.1	3	54430593	0.515
OAR26_21099683.1	26	17856378	0.515
OAR15_40325037.1	15	38495418	0.515
OAR10_90612987.1	10	83028244	0.515
OAR16_9870495.1	16	9003821	0.514
OAR3_92944291.1	3	87779593	0.514
OAR1_43083702.1	1	41602942	0.514
DU324205_588.1	2	232771356	0.514
s18566.1	15	54478418	0.514
s28047.1	15	31746519	0.514
OAR1_117165620.1	1	108366171	0.514
s32851.1	1	245923408	0.514
s23721.1	2	238643447	0.514
OAR15_28048435.1	15	26731567	0.514
s69711.1	9	14272267	0.514
OAR3_103689521.1	3	97441687	0.514
OAR6_43064935.1	6	38668794	0.514
OAR9_33331072.1	9	31817019	0.514
OAR15_38720759.1	15	36890254	0.513
OAR3_203583202.1	3	189046770	0.513
OAR4_21092552.1	4	20256712	0.513
OAR2_63781435.1	2	59306755	0.513
s65198.1	12	44498233	0.513
OAR8_60196419.1	8	56189320	0.513
OAR6_43034224.1	6	38639963	0.513
OAR26_9628606.1	26	7472379	0.513
OAR23_3005816.1	23	2744610	0.513
OAR15_26646294.1	15	25481842	0.513
OAR10_68163245.1	10	65948793	0.512
OAR7_43593010.1	7	39394100	0.512
OAR7_68548573.1	7	62691903	0.512
s37611.1	2	245016588	0.512
OAR2_117650043.1	2	109394432	0.512
s12396.1	2	110554687	0.512
OAR5_93678808.1	5	85470418	0.511
OAR5_105926150.1	5	97354788	0.511
OAR16_42172047.1	16	38862399	0.510
s51748.1	5	59300808	0.510
OAR5_30620006_X.1	5	27593189	0.510
s19236.1	3	183135436	0.510

OAR1_260939703.1	4	106932088	0.510
OAR1_149631902.1	1	138506294	0.510
OAR2_221524097.1	2	209259024	0.510
s61002.1	16	17460862	0.510
s43611.1	1	107965473	0.510
s21965.1	11	12580028	0.509
OAR5_80804886.1	5	73554040	0.509
OAR1_16480770.1	1	16474302	0.509
OAR1_279502546.1	1	258589935	0.509
s30117.1	22	18619609	0.508
OAR16_37770793_X.1	16	34690414	0.508
OAR3_135687490.1	3	127211233	0.508
s17221.1	9	84920003	0.508
OAR16_27149060.1	16	24977627	0.508
OAR6_64800913.1	6	58816416	0.508
s73063.1	7	82851545	0.508
OAR2_40373894.1	2	38795830	0.508
OAR3_45774145.1	3	42749955	0.508
OAR1_71610348.1	1	67018785	0.507
OAR11_18701428.1	11	18325488	0.507
OAR11_18711856.1	11	18335747	0.507
OAR22_38946909.1	22	34121320	0.507
OAR12_62615870.1	12	56261324	0.507
OAR14_20057933.1	14	19647418	0.506
OAR1_156875899_X.1	1	145339557	0.506
s15682.1	25	41768454	0.506
s32813.1	7	39390045	0.506
OAR3_84882715.1	3	80279564	0.506
OAR1_106884058.1	1	99602130	0.506
OAR1_232191666.1	1	215174264	0.506
OAR19_1651207.1	19	1632692	0.506
OAR1_245730132.1	1	228049380	0.506
OAR2_19500065.1	2	19163821	0.505
OAR6_45004760.1	6	40325304	0.505
OAR2_65702051.1	2	61245971	0.505
s37564.1	3	165413630	0.505
OAR12_30481802.1	12	26976828	0.505
s33649.1	4	103224760	0.505
OAR2_215636399.1	2	203788683	0.505
OAR3_122249804.1	3	114691829	0.505
OAR1_117951301.1	1	109177873	0.505
s39245.1	4	113410788	0.505
s23284.1	3	37747759	0.505
OAR2_41944715.1	2	40269973	0.505

s31685.1	22	43182266	0.504
s04872.1	3	77787703	0.504
s45361.1	6	113208301	0.504
OAR5_26029786.1	5	23210050	0.504
OAR16_37779529.1	16	34679175	0.504
OAR7_61660987.1	7	55726352	0.504
OAR2_146752433.1	2	138151463	0.504
OAR23_49228531_X.1	23	46500641	0.503
OAR13_8755501.1	13	7768983	0.503
OAR2_42759240.1	2	40923200	0.503
OAR10_28029657.1	10	28014277	0.503
OAR3_233561444.1	3	215487255	0.503
s10838.1	13	65840975	0.503
OAR3_232198069.1	3	214218467	0.503
s36409.1	23	2462202	0.502
s46459.1	2	138226568	0.502
OAR1_52607477.1	1	50465210	0.502
OAR8_44684641.1	8	41478607	0.502
OAR1_206274474.1	1	191152073	0.502
OAR8_58842830.1	8	54911849	0.502
s20082.1	19	56296821	0.502
s06021.1	1	45070786	0.502
s08299.1	7	42907174	0.502
OAR6_120433331.1	6	105984644	0.502
OAR14_39834952.1	14	38250883	0.501
s67459.1	12	75057710	0.501
OAR2_145109349.1	2	136442416	0.501
OAR3_127833064.1	3	120032144	0.501
s23053.1	12	63448289	0.501
s55111.1	1	260284248	0.501
s41467.1	26	36138131	0.501
s48830.1	1	68061632	0.500
OAR1_245583569.1	1	227903221	0.500
OAR4_96338094.1	4	90764708	0.500
OAR2_91750500.1	2	86266274	0.500
OAR12_16257610.1	12	13596021	0.500
s28736.1	2	13932966	0.500
OAR6_6343216.1	6	4306619	0.500
OAR13_8168026.1	13	7214961	0.500
s38656.1	19	43212090	0.500
OAR17_35388997.1	17	32435431	0.500
OAR1_45896510.1	1	44322623	0.499
OAR19_23119299.1	19	21996583	0.499
OAR1_232161366.1	1	215145368	0.499

s36709.1	5	97322182	0.499
OAR3_4733096.1	3	4737569	0.499
OAR17_28687373.1	17	26152386	0.499
OAR2_141546800.1	2	133040292	0.499
s42756.1	6	868868	0.499
OAR11_34497217.1	11	32274192	0.499
OAR26_35926259.1	26	31588369	0.499
s59649.1	20	15613020	0.499
OAR9_32928388.1	9	31436214	0.499
OAR1_81125140.1	1	75937545	0.499
OAR1_221226809.1	1	204866048	0.498
s17646.1	3	90772017	0.498
s07311.1	17	17030366	0.498
OAR1_52398580.1	1	50250861	0.498
s30198.1	26	26347958	0.498
s11164.1	4	70385766	0.498
OAR9_60143990.1	9	57228393	0.498
OAR13_72735047.1	13	67561757	0.498
DU296930_365.1	7	44204454	0.498
OAR9_82857185.1	9	78159432	0.498
s36390.1	11	41680008	0.498
s27301.1	6	23912970	0.497
OAR3_27655471.1	3	25602551	0.497
OAR9_79410987.1	9	74839637	0.497
OAR13_73111137.1	13	67942670	0.497
OAR10_45769189.1	10	45184017	0.497
OAR2_133088440.1	2	124907852	0.497
s09574.1	6	69061103	0.497
OAR6_37459560.1	6	33467474	0.497
s30707.1	25	14961423	0.497
s73318.1	3	103893158	0.497
OAR1_144742152.1	1	133776177	0.497
s32962.1	11	61580519	0.496
OAR5_112273600.1	5	103136478	0.496
OAR3_152889025.1	3	143046737	0.496
s21001.1	26	23065510	0.496
OAR8_84878537.1	8	78705571	0.496
OAR6_19924257.1	6	17008003	0.496
s35810.1	1	90630943	0.496
s43383.1	24	38985778	0.496
OAR2_61169025.1	2	56880389	0.495
DU317902_536.1	8	10705915	0.495
OAR14_33142743.1	14	31832814	0.495
OAR6_45040786.1	6	40356705	0.495

OAR7_43027191.1	7	38944126	0.495
s73474.1	10	85695176	0.495
OAR4_86671539.1	4	81579878	0.495
s61390.1	25	29623004	0.495
OAR3_196506344.1	3	182497551	0.495
OAR12_85001046.1	12	77094271	0.495
OAR4_11339554.1	4	11195774	0.495
OAR10_49074172.1	10	48300368	0.495
OAR9_1497546.1	9	1776818	0.495
OAR2_141764296.1	2	133272817	0.494
OAR9_7003718.1	9	7145715	0.494
s12590.1	18	20040105	0.494
s15191.1	16	39007570	0.494
s05053.1	17	63298962	0.494
s07518.1	5	58793978	0.494
OAR1_156775749.1	1	145235658	0.494
OAR17_75701731.1	17	69462601	0.493
OAR5_98944989.1	5	90712035	0.493
s10292.1	25	41750825	0.493
s23007.1	12	13495876	0.493
s57470.1	24	1938133	0.493
s29690.1	5	36496148	0.493
OAR5_108434752.1	5	99559078	0.493
s16964.1	13	30656334	0.493
OAR1_109602753.1	1	101965756	0.493
OAR1_51923225.1	1	49788536	0.493
OAR1_279560752.1	1	258640124	0.493
OAR6_101291449.1	6	92319183	0.493
OAR15_77962165.1	15	71926872	0.493
OAR3_179813294.1	3	167375732	0.493
OAR2_66116376.1	2	61657597	0.493
s29854.1	5	79322514	0.493
s18119.1	9	32284404	0.493
OAR7_55075040.1	7	49719850	0.493
OAR2_124173700.1	2	116166895	0.493
OAR1_281649480.1	1	260317241	0.492
OAR14_19829145.1	14	19407789	0.492
OAR13_17030546_X.1	13	11865807	0.492
OAR3_203907310.1	3	189329851	0.492
s66799.1	21	24578652	0.492
OAR4_80710186.1	4	76106568	0.492
OAR17_9725212.1	17	8762592	0.492
OAR3_98857686.1	3	93150623	0.492
s51903.1	17	63961031	0.492

DU381045_479.1	14	61164458	0.492
s52618.1	5	59485810	0.492
OAR8_55750248.1	8	51902877	0.491
OAR9_30026380.1	9	28723219	0.491
OAR16_43196533.1	16	39713867	0.491
OAR5_112227256.1	5	103087994	0.491
OAR3_92904936.1	3	87747902	0.491
OAR5_94755795.1	5	86658080	0.491
OAR1_45270297.1	1	43681990	0.491
s42972.1	1	261414809	0.490
OAR4_114665630.1	4	107206095	0.490
OAR9_1473217.1	9	1751809	0.490
OAR25_31570574.1	25	30210216	0.490
OAR3_152509773_X.1	3	142676994	0.490
OAR10_28385909.1	10	28371400	0.490
s32565.1	5	36059582	0.490
OAR1_220545599_X.1	1	204205759	0.490
s39632.1	8	19596761	0.490
OAR26_14501727.1	26	11785652	0.489
OAR1_124151951.1	1	115131726	0.489
s67786.1	12	25049404	0.489
OAR15_37813948.1	15	35973111	0.489
OAR24_8719574.1	24	7483090	0.489
s18948.1	7	44258658	0.489
OAR2_65907499.1	2	61449429	0.489
s71258.1	17	16987281	0.489
OAR2_34308093.1	2	32995715	0.489
s63142.1	13	32868564	0.489
OAR19_23147477.1	19	22023369	0.489
s47846.1	1	243372009	0.489
s22354.1	11	47578629	0.489
OAR11_34637871.1	11	32411650	0.488
OAR4_59938222.1	4	56746895	0.488
s25959.1	11	30692319	0.488
s28814.1	3	103477989	0.488
OAR19_30535417.1	19	28943148	0.488
OAR26_29804026.1	26	25738479	0.488
OAR15_32949553.1	15	31492057	0.488
OAR15_20741447.1	15	19935052	0.488
OAR4_50013760.1	4	47296741	0.488
OAR16_48444618.1	16	44501487	0.488
OAR4_68712576.1	4	64957575	0.487
OAR13_26732874.1	13	24150509	0.487
s03044.1	21	45197021	0.487

s04530.1	3	217143245	0.487
OAR1_72635280.1	1	68118567	0.486
OAR2_128835133.1	2	120501335	0.486
s04158.1	1	227782846	0.486
s68471.1	2	13928073	0.486
OAR4_13235164.1	4	13051653	0.486
OAR6_101835781.1	6	92817046	0.486
OAR2_203533603.1	2	192149660	0.485
OAR25_13459673.1	25	13170473	0.485
OAR3_95910669.1	3	90308553	0.485
OAR9_33550471.1	9	32025839	0.485
OAR1_181938531.1	1	168716279	0.485
OAR22_42978165.1	22	38194425	0.485
s51831.1	9	78451353	0.485
s51574.1	21	42278850	0.485
OAR3_84974488.1	3	80369355	0.485
OAR19_17697790.1	19	16934497	0.485
s27767.1	22	18649988	0.485
OAR10_30059832.1	10	30025043	0.485
OAR1_245412211.1	1	227804584	0.484
s62569.1	3	7182050	0.484
OAR2_95733865.1	2	89239384	0.484
OAR16_33136420.1	16	30477701	0.484
s28909.1	2	136485296	0.484
OAR6_111041196.1	6	100919997	0.484
OAR13_2388727.1	13	1861848	0.483
OAR2_245879797.1	2	232791702	0.483
OAR16_13527056.1	16	12453059	0.483
OAR8_86672870.1	8	80423696	0.483
OAR7_50086827.1	7	45356120	0.483
s06234.1	1	262938591	0.483
OAR7_90352641.1	7	82979915	0.483
OAR14_58084909.1	14	54913688	0.483
s62669.1	13	64345635	0.483
s75402.1	19	45399607	0.483
OAR3_16713871.1	3	15482582	0.483
OAR13_38550737.1	13	35153816	0.483
s55525.1	5	88367189	0.483
s05468.1	15	31807894	0.483
s48689.1	5	17065689	0.483
OAR23_2188429.1	23	1946410	0.483
s71629.1	1	27398178	0.482
OAR10_84355226.1	10	77142109	0.482
OAR2_143195752.1	2	134667278	0.482

OAR3_27713287.1	3	25659112	0.482
s13245.1	23	31161678	0.482
s48633.1	18	21691344	0.482
OAR2_60738297.1	2	56446750	0.482
OAR4_93235191.1	4	87800446	0.482
OAR2_141513613.1	2	133009157	0.482
OAR1_52767848.1	1	50621736	0.481
OAR2_66195373.1	2	61735560	0.481
OAR3_79055518.1	3	74855304	0.481
OAR6_15450053.1	6	12892260	0.481
OAR2_145075673.1	2	136412426	0.481
OAR23_32570581.1	23	30889404	0.481
OAR19_31737971_X.1	19	30098502	0.481
OAR15_8363565.1	15	8651422	0.481
s27554.1	14	38306508	0.481
OAR16_37614079.1	16	34625175	0.481
OAR2_3394232.1	2	4556476	0.480
s33128.1	3	104217317	0.480
s01940.1	2	23277528	0.480
OAR26_49379510.1	26	43479991	0.480
s33596.1	3	4658152	0.480
OAR1_265606095.1	1	245969875	0.480
OAR8_80619619.1	8	74682690	0.479
OAR8_87046223.1	8	80774629	0.479
OAR22_12905080.1	22	10962391	0.479
s00040.1	15	27235356	0.479
OAR23_38260590.1	23	36185015	0.479
s62496.1	2	14402382	0.479
OAR15_13190017_X.1	15	13177045	0.479
s28171.1	7	40030408	0.479
s03280.1	25	40741284	0.479
OAR22_29227333.1	22	24908175	0.479
s46527.1	13	68404235	0.479
OAR5_87366264.1	5	79478908	0.479
OAR15_28910424.1	15	27591341	0.479
s30085.1	3	80164042	0.479
s19977.1	9	90540171	0.479
OAR2_243336495.1	2	230359001	0.479
s42036.1	7	73758940	0.478
OAR3_22309460.1	3	20643497	0.478
s12848.1	2	191834956	0.478
s62290.1	3	97382388	0.478
OAR2_118492152.1	2	110284541	0.478
OAR6_55564892.1	6	50257985	0.478

s25228.1	15	31291443	0.478
s67403.1	3	104001939	0.478
s25920.1	8	80872789	0.478
OAR14_16764006.1	14	16283237	0.478
OAR22_45546460.1	22	40518236	0.478
s03597.1	6	113440194	0.478
OAR8_57889261.1	8	53999261	0.478
s64801.1	18	21628895	0.478
OAR5_62692616.1	5	57289343	0.478
s46498.1	17	69517635	0.477
s27209.1	12	68444880	0.477
s30564.1	8	28037083	0.477
OAR3_226870159.1	3	209206117	0.477
s47031.1	22	49622964	0.477
OAR8_18067475.1	8	16186584	0.477
s51441.1	19	13577090	0.477
OAR4_21244142.1	4	20398963	0.477
OAR1_166085590.1	1	154275654	0.477
OAR2_143949087.1	2	135337045	0.476
OAR1_245352370.1	1	227745997	0.476
OAR9_6578440.1	9	6652961	0.476
OAR1_124002420.1	1	115091200	0.476
OAR3_162494508.1	3	152029515	0.476
OAR6_91229780.1	6	83488418	0.476
OAR2_77016276.1	2	72254024	0.476
s62140.1	1	185546271	0.476
OAR2_86617792.1	2	81452694	0.475
OAR23_32059301.1	23	30537717	0.475
s53274.1	1	7748239	0.475
OAR1_265516050.1	1	245879353	0.475
OAR2_144406868.1	2	135796714	0.475
s40291.1	11	41530998	0.475
OAR13_41982854.1	13	38520473	0.475
s31212.1	23	20160121	0.475
s49070.1	1	109251246	0.475
OAR3_95548607.1	3	90046082	0.475
s16604.1	24	20005048	0.475
OAR1_52908583.1	1	50766596	0.475
OAR16_15512379.1	16	14273045	0.474
OAR6_568937.1	6	813227	0.474
OAR3_78128087.1	3	73964191	0.474
s21107.1	25	7618495	0.474
OAR1_53726695.1	1	51449360	0.474
s19550.1	4	47483537	0.474

OAR4_50210185.1	4	47486423	0.474
s57405.1	2	124467465	0.474
s23854.1	10	13428227	0.474
OAR6_91476645.1	9	60802870	0.474
s61506.1	9	32003055	0.474
s36620.1	5	59507633	0.474
OAR8_33710104.1	8	30994440	0.473
OAR18_33027908.1	18	31650188	0.473
s24134.1	11	47885965	0.473
OAR25_32156972.1	25	30802961	0.473
s33774.1	7	73731645	0.473
s42653.1	3	104154438	0.473
OAR1_149763555.1	1	138616449	0.473
OAR15_76144996.1	15	70199575	0.473
OAR1_198827329.1	1	184250056	0.473
s46479.1	16	8968749	0.473
OAR5_104118989.1	5	95589703	0.473
s25195.1	25	7392689	0.473
s08622.1	1	251795181	0.473
OAR2_20784358.1	2	20379763	0.473
s34980.1	10	27398550	0.473
OAR16_33253515.1	16	30589279	0.472
OAR10_12067705.1	10	13509048	0.472
s16015.1	1	100001324	0.472
OAR10_86074123.1	10	78849033	0.472
OAR24_42974369.1	24	39757470	0.472
s00503.1	2	61227472	0.472
OAR8_22108137.1	8	19611240	0.472
s30686.1	22	40258358	0.472
OAR1_72776475.1	1	68250822	0.472
OAR8_27294787.1	8	24860110	0.472
OAR9_8215268.1	9	8311300	0.472
OAR7_77829558.1	7	71010039	0.472
s42703.1	25	17652415	0.472
OAR2_92655139.1	2	87113994	0.472
OAR25_31530072_X.1	25	30169926	0.472
s67777.1	7	20716990	0.472
s42921.1	15	64119066	0.472
OAR3_15534872.1	3	14388844	0.472
OAR15_13695309.1	15	13680245	0.472
OAR3_57489213.1	3	54368964	0.471
s05399.1	15	38530749	0.471
OAR7_44587319.1	7	40270425	0.471
s43298.1	21	30695655	0.471

s03860.1	7	42006386	0.471
OAR2_213278806.1	2	201514161	0.471
OAR6_47541995.1	6	42591389	0.471
s09113.1	19	44286116	0.471
OAR3_235332945.1	3	217195979	0.471
s08824.1	20	39776900	0.471
DU397126_450.1	14	38330512	0.470
s70994.1	19	16888900	0.470
s32734.1	19	45854502	0.470
OAR7_100185924.1	7	91993631	0.470
s53959.1	26	7435938	0.470
OAR11_12207260.1	11	12456595	0.470
OAR22_29129826.1	22	24803666	0.470
s55016.1	13	80920190	0.470
OAR1_52004573.1	1	49870684	0.470
OAR14_54195253.1	14	51284257	0.470
OAR4_82770525.1	4	78021185	0.470
OAR16_33519318.1	16	30886595	0.470
OAR20_18651357.1	20	17800631	0.470
OAR2_64231337.1	2	59762935	0.470
OAR3_98987819.1	3	93260015	0.470
OAR3_125653475.1	3	117778341	0.470
OAR8_83458866.1	8	77347407	0.470
DU299931_445.1	2	81407809	0.470
OAR22_22410821.1	22	18541303	0.470
OAR14_27519100.1	14	26343076	0.470
s47338.1	1	98211805	0.469
s05350.1	3	103599900	0.469
OAR1_156714280.1	1	145180382	0.469
OAR16_15458830.1	16	14215465	0.469
OAR8_12209748.1	8	10901870	0.469
s36267.1	5	88338774	0.469
OAR2_66083138.1	2	61621077	0.469
s27329.1	23	56674506	0.469
s62976.1	5	59530500	0.469
OAR2_176738567_X.1	2	167096123	0.469
OAR5_108561009_X.1	5	99641244	0.469
s52058.1	7	17834128	0.469
s23821.1	13	60895517	0.469
OAR23_32551191.1	23	30870894	0.468
OAR2_180985316.1	2	170652224	0.468
OAR18_10826154.1	18	10853032	0.468
OAR5_96757423.1	5	88720507	0.468
OAR11_51856858.1	11	48848474	0.468

OAR8_62334652.1	8	57996793	0.468
OAR7_46559347.1	7	41967369	0.468
OAR10_57284787.1	10	56149162	0.468
OAR1_194938697.1	1	180609768	0.468
OAR8_90480980.1	8	83890608	0.468
s21038.1	19	53584885	0.468
OAR5_98847734.1	5	90613401	0.468
OAR2_91638206.1	2	86164251	0.468
OAR10_85485865.1	10	78296997	0.468
s65835.1	22	18684922	0.468
OAR2_239828382.1	2	227072901	0.468
s01546.1	7	59940866	0.468
OAR14_16713218.1	14	16236771	0.468
s47519.1	2	97741243	0.468
OAR6_101315163.1	6	92343909	0.467
OAR8_36508728.1	8	33855818	0.467
OAR7_30442652.1	7	26745110	0.467
OAR3_89860596.1	3	84911759	0.467
OAR23_58632679.1	23	55141170	0.467
OAR1_105078097.1	1	98412220	0.467
OAR3_92868792.1	3	87710569	0.467
OAR6_22138611.1	6	19161699	0.467
s08174.1	3	65177707	0.467
OAR3_152114001.1	3	142284913	0.467
OAR1_86080692.1	1	80801999	0.467
s11162.1	17	21145424	0.467
s10394.1	6	69005883	0.467
OAR2_165417013.1	2	156001345	0.467
s39748.1	17	65576794	0.467
OAR6_6211452.1	6	4193393	0.467
OAR9_99753311.1	9	93740939	0.467
OAR3_233491871.1	3	215419146	0.466
OAR2_205138896.1	2	193596846	0.466
OAR8_31201923.1	8	28788770	0.466
OAR8_67978586.1	8	63186794	0.466
OAR3_138246433.1	3	129604964	0.466
OAR5_94915332.1	5	86810078	0.466
s13775.1	12	61273373	0.466
s15084.1	7	82932447	0.466
OAR16_42184944.1	16	38878086	0.466
s42786.1	16	38880548	0.466
OAR2_132080120.1	2	123857066	0.466
OAR1_144817903.1	1	133906203	0.466
OAR4_93903479.1	4	88486000	0.466

OAR8_14152236_X.1	8	12602136	0.466
OAR16_40846568_X.1	16	37622186	0.466
OAR7_68394178.1	7	62539269	0.466
OAR4_27894204.1	4	26488638	0.465
OAR12_42878639.1	12	38475442	0.465
OAR2_155715202.1	2	146734798	0.465
s60265.1	12	50581422	0.465
OAR5_95001131.1	5	86902391	0.465
OAR21_13268403.1	21	11717379	0.465
s62814.1	2	191910639	0.465
OAR8_13942037.1	8	12411195	0.465
OAR2_128931922.1	2	120609452	0.465
OAR17_33466679.1	17	30581082	0.465
OAR9_32911813.1	9	31421304	0.465
OAR1_27632403.1	1	27287535	0.465
OAR2_237163960.1	2	224438092	0.464
OAR18_58942764.1	18	55155983	0.464
OAR2_63699523.1	2	59231945	0.464
OAR3_82364808.1	3	77832563	0.464
OAR1_198733751.1	1	184155995	0.464
s14823.1	19	15080853	0.464
OAR15_88109218.1	15	79199919	0.464
OAR8_76247205.1	8	71065708	0.464
OAR9_6755588.1	9	6877914	0.464
s58654.1	4	118489003	0.464
OAR2_147550458.1	2	138951962	0.464
s64015.1	23	46368708	0.463
s00904.1	9	1631021	0.463
OAR26_21018684.1	26	17819004	0.463
OAR6_549040.1	6	795947	0.463
s69644.1	17	8831140	0.463
OAR7_53497046.1	7	48439913	0.463
OAR1_60333122.1	1	57316101	0.463
s14360.1	4	115058153	0.463
OAR4_96389676.1	4	90818623	0.463
OAR5_105813966.1	5	97246551	0.463
OAR6_51132829.1	6	46119077	0.463
OAR15_76674101.1	15	70762081	0.463
s18172.1	6	76487965	0.462
OAR23_50421087.1	23	47553083	0.462
OAR8_67122251.1	8	62326453	0.462
s13888.1	13	67622358	0.462
OAR3_125733929.1	3	117854395	0.462
OAR23_9819232.1	23	8919709	0.462

OAR7_47757823.1	7	43098726	0.462
OAR23_13573358.1	23	12431055	0.462
s56913.1	15	54724620	0.462
OAR2_23837222.1	2	23487206	0.462
OAR3_152786161.1	3	142944347	0.462
OAR4_96250480.1	4	90675504	0.462
OAR6_109205577.1	6	99193263	0.462
s00799.1	4	117189277	0.462
s19983.1	3	93879267	0.461
s01154.1	6	45580772	0.461
OAR12_47193127.1	12	42399721	0.461
OAR17_23630662.1	17	21221285	0.461
OAR9_83083144.1	9	78385677	0.461
OAR2_47698708.1	2	45015658	0.461
s61648.1	12	1263847	0.460
s10803.1	6	109626423	0.460
OAR16_37419505.1	16	34392335	0.460
OAR23_2760770.1	23	2494257	0.460
s42931.1	13	80479855	0.460
OAR11_3642255.1	11	4256308	0.460
OAR6_37483582.1	6	33493183	0.460
OAR20_46608631.1	20	42923555	0.460
OAR21_47882305.1	21	43296505	0.460
OAR4_62240280.1	4	58920751	0.460
OAR4_92808545.1	4	87392051	0.460
OAR8_13873014.1	8	12342949	0.460
OAR5_55048051.1	5	50759855	0.460
OAR18_10963695.1	18	10984471	0.460
OAR5_23734357.1	5	21094473	0.460
OAR13_26553208.1	13	24023458	0.460
OAR6_24843953.1	6	21628996	0.460
s08197.1	25	40509990	0.460
OAR2_148534584.1	2	139718962	0.460
s26118.1	7	73668292	0.460
OAR8_91660520.1	8	84997173	0.460
OAR6_6157042.1	6	4155782	0.459
OAR26_5770772.1	26	5131055	0.459
OAR16_72384133.1	16	66579927	0.459
s02671.1	13	65458747	0.459
OAR2_132019304.1	2	123794330	0.459
OAR14_39926553.1	14	38342841	0.459
OAR7_54141447.1	7	49042680	0.459
OAR7_17872303.1	7	17277451	0.459
OAR22_48686242.1	22	43334957	0.459

s29856.1	5	59105733	0.459
OAR2_60823920.1	2	56521561	0.459
OAR9_32819540.1	9	31387692	0.459
s64219.1	6	68696291	0.459
OAR14_57968471.1	14	54802973	0.459
OAR1_229636965.1	1	212888343	0.459
OAR8_96572418_X.1	8	89585108	0.458
s57793.1	7	98610524	0.458
s31327.1	1	262960057	0.457
s31340.1	15	72536427	0.457
OAR5_83843279.1	5	76233696	0.457
s33178.1	16	38899653	0.457
s32200.1	23	12350788	0.457
OAR21_13608697.1	21	11968338	0.457
OAR11_11180584.1	11	11593659	0.457
OAR1_175040095.1	1	162284804	0.457
OAR8_12237843.1	8	10929938	0.457
OAR26_30036643.1	26	25957009	0.457
s74514.1	2	139455644	0.457
OAR2_157494688.1	2	148526445	0.457
OAR2_14765360.1	2	15322091	0.457
OAR22_32948290.1	22	28642922	0.456
s46756.1	1	16465248	0.456
OAR1_117214017.1	1	108415563	0.456
OAR5_94841254.1	5	86740574	0.456
OAR1_79619162.1	1	74416132	0.456
OAR1_197390718.1	1	182941584	0.456
s03792.1	2	132802249	0.456
s70896.1	3	60088420	0.456
OAR16_70695201_X.1	16	64971385	0.456
OAR14_16757677.1	14	16277597	0.456
OAR3_150564820.1	3	140760089	0.456
OAR4_100741190.1	4	95094427	0.456
OAR3_27771608.1	3	25710765	0.456
OAR4_46265640.1	4	43848244	0.456
OAR13_60550782.1	13	55660662	0.456
OAR14_53406640.1	14	50504964	0.456
OAR9_82085786.1	9	77473891	0.455
OAR12_43257167.1	12	38843578	0.455
OAR3_96170847.1	3	90549311	0.455
OAR26_16139285.1	26	13333552	0.455
OAR1_52374321.1	1	50229647	0.455
s73482.1	23	46624328	0.455
s62771.1	18	55537048	0.455

s53281.1	5	23299118	0.455
OAR2_32564484.1	2	31388914	0.455
OAR8_85517334.1	8	79239878	0.455
OAR1_200228185.1	23	36481538	0.455
OAR21_50331592.1	21	45332006	0.455
OAR2_66155982.1	2	61697022	0.455
OAR5_105969630.1	5	97402781	0.455
s33676.1	16	66300344	0.455
OAR12_46545889.1	12	41736967	0.455
s24872.1	7	15988141	0.455
OAR10_49013559.1	10	48243886	0.455
OAR1_117978374.1	1	109210933	0.455
s42531.1	4	71870600	0.454
OAR4_100777666.1	4	95130608	0.454
s32466.1	16	9124681	0.454
OAR10_71907148.1	10	69516727	0.454
s09080.1	1	94310141	0.454
s74913.1	12	44629062	0.454
OAR2_221490831.1	2	209225353	0.454
OAR4_114714505.1	4	107255476	0.454
OAR12_28746512.1	12	25268304	0.454
OAR4_100712500.1	4	95065514	0.453
OAR1_117268913.1	1	108471215	0.453
OAR18_62399925.1	18	58389719	0.453
s38891.1	2	233019342	0.453
OAR3_80043937.1	3	75748976	0.453
OAR10_22164858.1	10	22540345	0.453
OAR26_40962143.1	26	36031341	0.453
OAR3_150637864.1	3	140827769	0.453
s35389.1	19	30535888	0.453
OAR2_172741598.1	2	163109337	0.453
s48420.1	17	65744982	0.453
OAR2_66203050.1	2	61743310	0.453
s30831.1	1	176042371	0.453
OAR13_35982442.1	13	32625552	0.452
OAR5_96674157.1	5	88634982	0.452
OAR13_36521771.1	13	33160066	0.452
s60859.1	9	31944220	0.452
OAR22_48982599.1	22	43619480	0.452
s57130.1	4	24036246	0.452
s17757.1	11	8134841	0.452
s20463.1	21	29230702	0.452
s21984.1	13	81611070	0.452
s07075.1	12	56285258	0.452

OAR3_24844330.1	3	23079743	0.452
OAR11_50922447.1	11	47935334	0.452
OAR1_30673378.1	1	30096508	0.452
s61866.1	7	11801898	0.452
s46382.1	16	39157118	0.451
s39907.1	10	80450776	0.451
OAR15_22368903.1	15	21448285	0.451
OAR14_37445471.1	14	35992007	0.451
OAR1_172390718.1	1	159827512	0.451
s44523.1	2	23355101	0.451
OAR2_177639830.1	2	167885360	0.451
OAR1_105916901.1	1	98698690	0.451
OAR8_89506288.1	8	82987574	0.451
OAR16_59388986.1	16	54486204	0.451
OAR13_16924382.1	13	11955869	0.451
OAR15_18385515_X.1	15	17824040	0.451
OAR2_75637263.1	2	70976797	0.451
OAR13_33896421.1	13	30733899	0.451
OAR1_107103247.1	1	99799572	0.451
s31260.1	14	39415649	0.451
s52289.1	9	16197331	0.450
OAR8_58215143.1	8	54310629	0.450
s35197.1	11	30193254	0.450
s17924.1	5	16729235	0.450
OAR5_94876397.1	5	86770859	0.450
OAR16_43833978.1	16	40339621	0.450
OAR8_50989547.1	8	47509991	0.450
OAR4_41399083.1	4	39299669	0.450
OAR23_61577887.1	23	57787391	0.450
s02221.1	15	13271015	0.450
s23190.1	2	248504893	0.450
OAR10_91658465.1	10	84029946	0.449
OAR2_180239034.1	2	170195998	0.449
OAR25_31629845.1	25	30273221	0.449
OAR5_104240083.1	5	95687342	0.449
s68251.1	2	170303370	0.449
OAR2_69526155.1	2	65040471	0.449
s18550.1	7	15995141	0.449
OAR19_17575750.1	19	16820490	0.449
OAR1_140939577.1	1	130124668	0.449
OAR4_48684803.1	4	45967609	0.449
OAR18_20633775.1	18	20134921	0.449
s03306.1	2	2765814	0.449
OAR19_56538781_X.1	19	53499133	0.449

OAR3_170807307.1	3	159692296	0.449
s16744.1	15	24083295	0.449
OAR25_31677314.1	25	30315848	0.449
OAR6_55349679.1	6	50058344	0.449
OAR9_42902329.1	9	40868212	0.449
OAR16_42312325.1	16	38943467	0.449
OAR7_80500931.1	7	73648676	0.449
OAR3_207738289.1	3	193035777	0.449
s03705.1	25	40993152	0.449
OAR14_53138348.1	14	50238610	0.449
OAR4_100874235.1	4	95225295	0.448
OAR9_93788043.1	9	88282528	0.448
OAR3_169198367.1	3	158111043	0.448
s57119.1	5	50782236	0.448
s23222.1	12	40442151	0.448
s23129.1	14	6208692	0.448
OAR2_34301332.1	2	32988451	0.448
s64925.1	22	43571769	0.448
OAR1_145462895.1	1	134460874	0.448
OAR5_96703012.1	5	88661821	0.448
OAR1_42933481.1	1	41456605	0.448
OAR23_9715053.1	23	8805942	0.448
s51183.1	10	13338205	0.448
s61729.1	6	45439964	0.448
OAR2_166400154.1	2	156985451	0.448
s71447.1	1	29110758	0.447
s72656.1	9	93799321	0.447
OAR3_150573902.1	3	140766356	0.447
s40920.1	17	28931348	0.447
OAR2_236249490.1	2	223460997	0.447
OAR16_27136698.1	16	24968600	0.447
OAR24_8989305.1	24	7833106	0.447
OAR3_92984654.1	3	87822190	0.447
OAR19_40904872.1	19	38977959	0.447
OAR8_27648193.1	8	25218113	0.447
s44871.1	6	6497783	0.447
s16440.1	7	43245323	0.447
OAR6_50222315.1	6	45296994	0.447
OAR15_11973263.1	15	12052518	0.447
s61859.1	25	31037322	0.446
DU352764_273.1	15	50813916	0.446
OAR7_1181460_X.1	7	1482274	0.446
OAR15_87912118.1	15	78941317	0.446
s55008.1	1	225356104	0.446

OAR15_67212591.1	15	61815728	0.446
OAR3_127680786.1	3	119882148	0.446
OAR5_40166532_X.1	5	36419513	0.446
OAR3_152083446.1	3	142253982	0.446
OAR25_13419677.1	25	13140616	0.446
OAR10_49548443.1	10	48775457	0.446
OAR8_18043643.1	8	16168861	0.446
OAR19_37314076_X.1	19	35573209	0.446
OAR16_9947075.1	16	9075249	0.446
OAR21_28535197.1	21	25559077	0.446
s71238.1	15	13649195	0.446
OAR1_297285976.1	1	274755523	0.446
OAR9_87478637.1	9	82682823	0.445
OAR1_228489740.1	1	211846418	0.445
OAR8_58181648_X.1	8	54280168	0.445
s28234.1	1	109063783	0.445
OAR12_49617704.1	12	44709450	0.445
OAR3_151929197.1	3	142109502	0.445
OAR8_27161632.1	8	24707933	0.445
DU461176_154.1	1	99896856	0.445
OAR1_65028926.1	1	61397709	0.445
s41022.1	2	32893038	0.445
s32732.1	2	127997234	0.445
OAR24_42952578.1	24	39735778	0.445
OAR15_87315790.1	15	78695106	0.445
OAR20_46680243.1	20	43001737	0.445
OAR3_232125965.1	3	214149668	0.445
OAR6_91292213.1	6	83562872	0.445
OAR26_36231458.1	26	31875529	0.445
s61320.1	3	7192808	0.445
OAR2_12469561.1	2	13078663	0.445
s05045.1	24	32800970	0.444
OAR6_26128546.1	6	22846685	0.444
OAR16_31817206.1	16	29276425	0.444
OAR17_9821235.1	17	8860722	0.444
s73069.1	19	56309709	0.444
OAR19_46545362.1	19	44209101	0.444
OAR22_43835577.1	22	38924538	0.444
OAR3_45394131.1	3	42398511	0.444
OAR13_70805821.1	13	65720624	0.444
OAR25_43096371.1	25	40766187	0.444
s08584.1	2	230323317	0.444
OAR1_79688436.1	1	74482904	0.444
OAR18_26087486.1	18	25173878	0.444

OAR12_35168968.1	12	31357766	0.444
OAR1_245302998.1	1	227692672	0.443
s64023.1	5	16746930	0.443
s10057.1	17	60654434	0.443
s29197.1	2	149380990	0.443
OAR5_104210549.1	5	95657941	0.443
s47946.1	6	113881838	0.443
OAR23_64564298_X.1	23	60640087	0.443
OAR16_33630679.1	16	30990027	0.443
OAR2_153419559.1	2	144500078	0.443
OAR16_23680997.1	16	21654029	0.443
OAR2_165344428.1	2	155927328	0.443
OAR1_162623538.1	1	151186242	0.443
OAR8_60730321.1	8	56705959	0.443
OAR21_13212843.1	21	11660892	0.443
OAR26_36202800.1	26	31846749	0.443
s70624.1	7	21143650	0.443
OAR8_64834575.1	8	60187369	0.443
OAR5_75165666.1	5	68452787	0.443
OAR3_80182465_X.1	3	75884007	0.442
OAR10_10291004.1	10	11898594	0.442
s59454.1	2	45320994	0.442
OAR2_42769211.1	2	40931803	0.442
OAR2_165244995.1	2	155833687	0.442
s60284.1	9	16127970	0.442
OAR9_41187686.1	9	39227808	0.442
OAR1_44241542.1	1	42737432	0.442
OAR4_31128826.1	4	29612213	0.442
OAR1_29872607.1	1	29252531	0.442
OAR17_25364030.1	17	22962508	0.442
OAR3_134747538.1	3	126318550	0.442
OAR6_111022637.1	6	100900651	0.442
OAR3_152630913.1	3	142796592	0.442
s31264.1	17	28940958	0.442
OAR3_226847164.1	3	209183837	0.442
OAR6_44623765.1	6	39938674	0.442
OAR2_142646641.1	2	134194138	0.441
OAR6_67252446.1	6	61030506	0.441
OAR21_14165572.1	21	12497679	0.441
s18057.1	5	59379940	0.441
OAR20_49399263.1	20	45375105	0.441
OAR19_46813773.1	19	44497170	0.441
OAR1_52048533.1	1	49897092	0.441
OAR3_57464855.1	3	54343038	0.441

OAR1_52263906.1	1	50120343	0.441
OAR3_33093517.1	3	30928786	0.441
s41451.1	13	80946897	0.441
OAR26_41166096.1	26	36242897	0.441
OAR2_172802190.1	2	163170710	0.441
OAR11_11214325.1	11	11626457	0.441
OAR8_68261335.1	8	63473370	0.441
OAR3_19685356.1	3	18172749	0.441
s04976.1	5	16754839	0.441
OAR2_172603349.1	2	162861327	0.441
s03686.1	25	7517270	0.441
OAR4_11532952.1	4	11380946	0.441
OAR20_50828611.1	20	46721819	0.440
OAR19_10882333.1	19	10566691	0.440
OAR1_45835401.1	1	44262410	0.440
OAR3_93551207.1	3	88334095	0.440
OAR7_53605138.1	7	48549329	0.440
OAR25_43145095.1	25	40811951	0.440
OAR15_76321531.1	15	70393057	0.440
s33336.1	7	73552515	0.440
OAR6_19886420.1	6	16969882	0.440
s60824.1	17	63386591	0.440
OAR19_42032872.1	2	109369155	0.440
OAR10_22217205.1	10	22592412	0.440
s66980.1	18	59441058	0.440
OAR12_42546972.1	12	38183659	0.440
s05734.1	9	93860515	0.440
OAR22_10565085_X.1	22	8826614	0.440
OAR18_31974215.1	18	30706077	0.439
OAR1_197593478.1	1	183138836	0.439
OAR4_49971009.1	4	47250555	0.439
OAR8_48160033.1	8	44692215	0.439
s37245.1	10	84297326	0.439
OAR15_81048498.1	15	74752130	0.439
s53597.1	1	109095881	0.439
OAR3_53590166.1	3	50888555	0.439
OAR3_151903542.1	3	142083071	0.439
OAR3_170924636.1	3	159795155	0.439
OAR3_79278326.1	3	75052306	0.439
s07077.1	3	221505368	0.439
s28123.1	16	2423854	0.439
OAR10_24711066.1	10	24801456	0.439
s44288.1	22	49544771	0.439
s41716.1	1	2317262	0.439

s75594.1	8	72484617	0.438
s12247.1	23	2672488	0.438
OAR8_3874844.1	8	3585887	0.438
OAR18_32432472.1	18	31052392	0.438
OAR2_13874225.1	2	14459533	0.438
s39037.1	3	4382555	0.438
OAR11_36045417.1	11	33722495	0.438
OAR21_13084748.1	21	11522332	0.438
OAR6_21601091.1	6	18643578	0.438
OAR25_37588149.1	25	35798667	0.438
OAR22_48945901.1	22	43582702	0.438
s06116.1	1	101820263	0.438
OAR2_23807521.1	2	23460849	0.438
OAR23_32628025.1	23	30945890	0.438
s72805.1	15	27396963	0.438
s22326.1	7	21169136	0.438
OAR13_73958074.1	13	68764851	0.438
OAR1_45559516.1	1	44026375	0.438
OAR3_84222063.1	3	79661259	0.438
s61400.1	2	26600287	0.438
s20556.1	2	162869558	0.438
OAR1_7104917.1	1	7578311	0.438
OAR21_50378541.1	21	45368906	0.438
OAR8_68313375.1	8	63526260	0.438
OAR17_67050348.1	17	61492873	0.438
OAR6_44395905.1	6	39713559	0.438
OAR6_65181728.1	6	59199571	0.437
OAR3_149319006.1	3	139533299	0.437
OAR4_25328239.1	4	24165394	0.437
OAR12_28755835.1	12	25277611	0.437
OAR24_8655784.1	24	7564606	0.437
s30887.1	26	708531	0.437
OAR14_2036727.1	14	1558966	0.437
s43969.1	3	103380239	0.437
DU522140_165.1	15	34491899	0.437
OAR6_105017216.1	6	95681155	0.437
s72376.1	12	77206908	0.437
s71394.1	3	154790327	0.437
s13949.1	17	17005027	0.437
s23051.1	26	5223244	0.437
OAR17_9513319.1	17	8557131	0.437
OAR6_27236488.1	6	23853685	0.437
OAR4_48939856.1	4	46229465	0.437
OAR17_24080832.1	17	21571728	0.437

s51090.1	2	233988816	0.437
OAR9_4225646.1	9	4328654	0.436
OAR9_989389.1	9	1262953	0.436
OAR16_32336229.1	16	29759501	0.436
OAR1_64635180.1	1	61082391	0.436
OAR10_19965597.1	10	20482744	0.436
OAR3_79784642.1	3	75514641	0.436
OAR3_79793003.1	3	75519294	0.436
OAR1_244882421.1	1	227308647	0.436
OAR1_3066756.1	1	3839440	0.436
s72169.1	5	21030271	0.436
OAR18_49779319.1	18	46758665	0.436
s50496.1	22	18733992	0.436
s40052.1	7	54608877	0.436
OAR13_80941028.1	13	75240652	0.436
s34856.1	5	85338509	0.435
OAR26_14345999_X.1	26	11684825	0.435
s37981.1	23	46362141	0.435
OAR23_63000790.1	23	59097813	0.435
s25952.1	13	17962348	0.435
OAR3_195730138.1	3	181711513	0.435
OAR15_23429119.1	15	22506656	0.435
OAR25_6573369.1	25	6973999	0.435
OAR13_20943553.1	13	18571666	0.435
OAR16_43363065.1	16	39864135	0.435
s31181.1	2	61578504	0.435
s01043.1	2	138371374	0.435
OAR3_152425395.1	3	142599802	0.434
OAR3_195520988.1	3	181549208	0.434
s49313.1	19	44067296	0.434
s11647.1	18	31799928	0.434
OAR3_204945666.1	3	190346801	0.434
OAR12_46438220.1	12	41684287	0.434
OAR3_233296350.1	3	215226320	0.434
OAR7_48061232_X.1	7	43372429	0.434
OAR10_71855989.1	10	69465074	0.434
s20006.1	21	12424303	0.434
s60293.1	11	47717468	0.434
s69919.1	8	71118274	0.434
OAR4_74410532.1	4	70335607	0.434
OAR1_245443477.1	1	227831862	0.433
OAR6_98017695.1	6	89417212	0.433
OAR9_64800745_X.1	9	61661127	0.433
OAR7_90813398.1	7	83429307	0.433

s06722.1	3	14225212	0.433
OAR6_55321610.1	6	50026392	0.433
OAR8_44629587.1	8	41538911	0.433
s54366.1	13	32716917	0.433
s14318.1	3	217757756	0.433
OAR23_48878158.1	23	46167308	0.433
OAR13_74505755.1	13	69243196	0.433
OAR1_116671699.1	1	107866754	0.433
s58438.1	5	95280484	0.433
OAR15_69700846_X.1	15	64145404	0.433
OAR2_145478943.1	2	136780869	0.433
OAR19_2409205.1	19	2286216	0.433
OAR9_38221892.1	9	36285902	0.433
OAR2_41879685.1	2	40205877	0.433
OAR21_2979332.1	21	2297354	0.433
s16798.1	19	11145254	0.433
s35301.1	3	217881782	0.433
OAR2_215596955.1	2	203751286	0.432
s70130.1	5	50882591	0.432
OAR15_87285774.1	15	78663623	0.432
OAR3_79085072.1	3	74879524	0.432
s16895.1	5	23139577	0.432
OAR9_97923017_X.1	9	92020509	0.432

Table S4. Annotation of a SNP which both have high F_{ST} and XPEHH value

SNP-ID	Chromosome	Position	F_{ST} (Top 0.01)	XPEHH (Top 0.05)	Gene	SNP within gene
OAR1_45270297.1	1	43681990	0.291	0.491	DEPDC1	
s17708.1	1	53355068	0.243	0.570	AK5	AK5
s17708.1	1	53355068	0.243	0.570	ZZZ3	
s66349.1	1	68199001	0.289	0.521	TGFBR3	TGFBR3
s55347.1	1	183336939	0.443	0.813	GPR156	
OAR1_197916874.1	1	183420073	0.360	0.960	FSTL1	
OAR1_197916874.1	1	183420073	0.360	0.960	LRRC58	
DU464218_590.1	1	183575060	0.255	0.859	NDUFB4	
OAR1_249572898.1	1	231602195	0.246	0.634	ARHGEF26	ARHGEF26
OAR1_268122939.1	1	248275678	0.280	0.612	PRR23B	
OAR1_281649480.1	1	260317241	0.278	0.492	ZNF295	ZNF295
s42972.1	1	261414809	0.346	0.490	CRYAA	
s42972.1	1	261414809	0.346	0.490	U2AF1	U2AF1
OAR2_12469561.1	2	13078663	0.245	0.445	AKAP2	
OAR2_12469561.1	2	13078663	0.245	0.445	C9ORF152	
OAR2_40373894.1	2	38795830	0.243	0.508	DPYSL2	
OAR2_61008309.1	2	56714900	0.371	0.771	TLE4	TLE4
OAR2_63274739.1	2	58896602	0.245	0.541	GNA14	
OAR2_63274739.1	2	58896602	0.245	0.541	VPS13A	VPS13A
OAR2_63781435.1	2	59306755	0.260	0.513	PRUNE2	PRUNE2
OAR2_77016276.1	2	72254024	0.256	0.476	SLC1A1	
OAR2_142646641.1	2	134194138	0.270	0.441	CHN1	
OAR2_142646641.1	2	134194138	0.270	0.441	CHRNA1	
s44712.1	2	135753568	0.248	0.577	MLTK	
s44712.1	2	135753568	0.248	0.577	RAPGEF4	RAPGEF4
OAR2_155715202.1	2	146734798	0.266	0.465	DPP4	
OAR2_155715202.1	2	146734798	0.266	0.465	SLC4A10	SLC4A10
s44167.1	2	243153063	0.316	0.544	EPHA8	EPHA8
s44167.1	2	243153063	0.316	0.544	ZBTB40	
s08514.1	3	39851694	0.464	1.185	CNRIP1	
s08514.1	3	39851694	0.464	1.185	PLEK	
OAR3_42729807.1	3	39986491	0.317	1.618	PNO1	
OAR3_42729807.1	3	39986491	0.317	1.618	PPP3R1	PPP3R1
OAR3_42907435.1	3	40163832	0.422	1.828	WDR92	WDR92
OAR3_53590166.1	3	50888555	0.273	0.439	CTNNA2	CTNNA2
s19512.1	3	76037302	0.276	0.694	FOXN2	
OAR3_82409790.1	3	77879156	0.329	0.693	EPAS1	
OAR3_96170847.1	3	90549311	0.279	0.455	LTBP1	LTBP1
OAR3_98710538.1	3	93004742	0.378	0.752	ANKRD53	
OAR3_98710538.1	3	93004742	0.378	0.752	NAGK	NAGK

OAR3_98710538.1	3	93004742	0.378	0.752	PAIP2B	
OAR3_98710538.1	3	93004742	0.378	0.752	TEX261	
s67952.1	3	93522006	0.533	0.743	DYSF	DYSF
OAR3_138331159.1	3	129685397	0.262	0.624	MRPL42	
s71169.1	3	129759745	0.451	0.546	CRADD	
s71169.1	3	129759745	0.451	0.546	SOCS2	
OAR3_195730138.1	3	181711513	0.307	0.435	PKP2	PKP2
s54635.1	3	214112448	0.263	0.793	DDX17	
s54635.1	3	214112448	0.263	0.793	KCNJ4	
s54635.1	3	214112448	0.263	0.793	KDELR3	
OAR4_10748026.1	4	10609123	0.362	0.809	CCDC132	CCDC132
OAR4_25295648.1	4	24132337	0.313	0.702	MEOX2	MEOX2
OAR4_73786063.1	4	69811362	0.317	0.688	NFE2L3	NFE2L3
OAR4_91329005.1	4	85930010	0.332	0.761	FAM3C	FAM3C
s11336.1	4	87572495	0.349	0.583	SLC13A1	
s56763.1	5	15912332	0.357	0.759	ACSBG2	
s67679.1	5	16017146	0.325	1.212	RANBP3	
s67679.1	5	16017146	0.325	1.212	RFX2	
s29567.1	5	16203101	0.251	1.175	DUS3L	
s29567.1	5	16203101	0.251	1.175	PRR22	
s07332.1	5	16209393	0.300	1.217	HSD11B1L	
s07332.1	5	16209393	0.300	1.217	LONP1	LONP1
s07332.1	5	16209393	0.300	1.217	QIL1	
s07332.1	5	16209393	0.300	1.217	RPL36	
s07332.1	5	16209393	0.300	1.217	SAFB	
s07332.1	5	16209393	0.300	1.217	TMEM146	
s38680.1	5	16520048	0.295	0.874	PTPRS	
s16895.1	5	23139577	0.278	0.432	FBN2	FBN2
s07518.1	5	58793978	0.256	0.494	HMGXB3	
s07518.1	5	58793978	0.256	0.494	PDE6A	
s07518.1	5	58793978	0.256	0.494	SLC26A2	
s07518.1	5	58793978	0.256	0.494	TIGD6	
OAR5_87409839_X.1	5	79542631	0.320	0.814	ALDH1L2	ALDH1L2
OAR5_87409839_X.1	5	79542631	0.320	0.814	ATG10	ATG10
OAR5_87409839_X.1	5	79542631	0.320	0.814	ATP6AP1L	ATP6AP1L
OAR5_87409839_X.1	5	79542631	0.320	0.814	RPS23	
s61354.1	5	90586440	0.378	0.574	FAM172A	FAM172A
OAR6_67376317.1	6	61138606	0.405	0.532	SHISA3	
s14577.1	7	26755264	0.420	0.574	FMN1	FMN1
OAR7_47161420.1	7	42551974	0.313	0.772	ZNF609	ZNF609
OAR7_61660987.1	7	55726352	0.262	0.504	DMXL2	
OAR7_61660987.1	7	55726352	0.262	0.504	SCG3	
OAR8_51076568.1	8	47596948	0.319	0.703	MDN1	MDN1
OAR8_60234503.1	8	56216655	0.330	0.676	EPB41L2	EPB41L2

s14938.1	9	795580	0.358	0.677	KCNQ5	KCNQ5
OAR9_1718056.1	9	2004658	0.245	0.697	RIMS1	RIMS1
s66058.1	9	32100676	0.286	0.769	KIAA0146	KIAA0146
OAR9_38221892.1	9	36285902	0.398	0.433	SDR16C5	
OAR9_38221892.1	9	36285902	0.398	0.433	SDR16C6	
OAR9_82428531.1	9	77756891	0.333	0.745	VPS13B	VPS13B
OAR10_28149069.1	10	28136160	0.264	0.631	CFDP2	
OAR10_28149069.1	10	28136160	0.264	0.631	STARD13	STARD13
s71442.1	10	82994149	0.290	0.855	ABHD13	
s71442.1	10	82994149	0.290	0.855	LIG4	
s71442.1	10	82994149	0.290	0.855	TNFSF13B	
OAR11_3180902.1	11	3834100	0.278	0.569	RPL3	
OAR11_6228500.1	11	6725442	0.291	0.524	ANKFN1	ANKFN1
OAR11_18711856.1	11	18335747	0.538	0.507	EVI2A	
OAR11_18711856.1	11	18335747	0.538	0.507	EVI2B	
OAR11_18711856.1	11	18335747	0.538	0.507	OMG	
OAR11_18823250.1	11	18440783	0.464	0.534	NF1	NF1
s33957.1	11	41563065	0.261	0.611	ACLY	ACLY
s33957.1	11	41563065	0.261	0.611	CNP	
s33957.1	11	41563065	0.261	0.611	KLHL10	
s33957.1	11	41563065	0.261	0.611	KLHL11	KLHL11
s33957.1	11	41563065	0.261	0.611	TTC25	
s27209.1	12	68444880	0.405	0.477	RPS6KC1	
OAR13_2388727.1	13	1861848	0.338	0.483	PLCB4	PLCB4
OAR13_36331465.1	13	32978582	0.298	0.890	ZEB1	ZEB1
s39564.1	13	43797381	0.337	0.618	KLF6	
s18401.1	13	65660964	0.293	0.619	C20ORF132	C2ORF132
s18401.1	13	65660964	0.293	0.619	RBL1	
s18401.1	13	65660964	0.293	0.619	RPN2	
s46185.1	13	78420224	0.316	0.705	DPM1	
s46185.1	13	78420224	0.316	0.705	KCNG1	
s46185.1	13	78420224	0.316	0.705	MOCS3	
OAR14_39926553.1	14	38342841	0.248	0.459	DHX38	
OAR14_39926553.1	14	38342841	0.248	0.459	HP	HP
OAR14_39926553.1	14	38342841	0.248	0.459	PKD1L3	
OAR14_39926553.1	14	38342841	0.248	0.459	PMFBP1	
OAR14_39926553.1	14	38342841	0.248	0.459	TXNL4B	
OAR15_22404265.1	15	21482600	0.315	0.650	PPP2R1B	
OAR15_22404265.1	15	21482600	0.315	0.650	SIK2	SIK2
OAR15_53721488.1	15	49215124	0.332	0.572	OR52K1	
s31340.1	15	72536427	0.411	0.457	ALX4	
s31340.1	15	72536427	0.411	0.457	EXT2	EXT2
OAR16_27229721.1	16	25021431	0.276	0.662	ARL15	ARL15
OAR17_31561091.1	17	28790576	0.292	0.628	EIF1	

OAR17_31561091.1	17	28790576	0.292	0.628	PHF17	
OAR17_31561091.1	17	28790576	0.292	0.628	SFT2D1	
OAR17_44358460.1	17	41064820	0.292	0.561	HNRNPK	
OAR19_14489436.1	19	13918425	0.332	0.628	ULK4	ULK4
s32677.1	19	31833784	0.261	0.641	MITF	
OAR19_46690670.1	19	44350807	0.249	0.625	ARHGEF3	
OAR19_46690670.1	19	44350807	0.249	0.625	C3ORF63	C3ORF63
OAR19_46690670.1	19	44350807	0.249	0.625	CCDC66	
OAR19_60184770.1	19	56634864	0.307	0.593	PPARG	
OAR21_50378541.1	21	45368906	0.378	0.438	MTL5	
OAR21_50378541.1	21	45368906	0.378	0.438	PPP6R3	PPP6R3
OAR22_34323192.1	22	29843623	0.330	0.599	MXI1	
OAR22_34323192.1	22	29843623	0.330	0.599	SMNDC1	
s19461.1	22	39739539	0.362	0.616	WDR11	
OAR22_45509727.1	22	40482146	0.456	0.829	FGFR2	FGFR2
s64015.1	23	46368708	0.267	0.463	LOXHD1	
s64015.1	23	46368708	0.267	0.463	RNF165	
s42703.1	25	17652415	0.256	0.472	C10ORF107	
s42703.1	25	17652415	0.256	0.472	OR9Q1	OR9Q1
OAR25_32156972.1	25	30802961	0.268	0.473	DUSP13	
OAR25_32156972.1	25	30802961	0.268	0.473	SAMD8	SAMD8
OAR25_32156972.1	25	30802961	0.268	0.473	VDAC2	
s67284.1	25	40426700	0.247	0.566	WAPAL	
OAR26_16139285.1	26	13333552	0.246	0.455	STOX2	STOX2

Table S5. Annotation of a SNP which just have high F_{ST} value

SNP-ID	Chromosome	Position	F_{ST}	XPEHH	Gene	SNP within gene
OAR13_52482285.1	13	49006951	0.775	-	PPP1CC	
OAR13_52630089.1	13	49140884	0.745	-	MUTED	MUTED
OAR4_72720594.1	4	68754789	0.722	-	RPS18	
OAR3_165009241.1	3	154213690	0.671	-	MSRB3	MSRB3
OAR4_72568832.1	4	68601186	0.657	-	HIBADH	
OAR11_27654920.1	11	26421905	0.480	-	ALOX12	ALOX12
OAR11_27654920.1	11	26421905	0.480	-	BCL6B	
OAR11_27654920.1	11	26421905	0.480	-	CLEC10A	
OAR11_27654920.1	11	26421905	0.480	-	RNASEK	
OAR11_27654920.1	11	26421905	0.480	-	SLC16A11	
OAR11_27654920.1	11	26421905	0.480	-	SLC16A13	
OAR11_27654920.1	11	26421905	0.480	-	SOD1	
OAR4_73050615.1	4	69082247	0.466	-	SKAP2	
OAR6_92321965.1	6	84350628	0.392	-	UGT2B4	
OAR6_92321965.1	6	84350628	0.392	-	UGT2B7	UGT2B7
s58920.1	17	52092174	0.364	-	PITPNM2	
OAR10_37157671.1	10	36385864	0.350	-	ZMYM5	
OAR3_200888015.1	3	186673626	0.347	-	PTHLH	
OAR7_63342726.1	7	57353770	0.335	-	ATP8B4	ATP8B4
OAR1_130003540.1	1	119845352	0.328	-	ATP5O	
OAR1_130003540.1	1	119845352	0.328	-	ITSN1	
s12884.1	13	62857560	0.324	-	RALY	RALY
OAR17_67295616.1	17	61733069	0.310	-	CLDN6	
OAR17_67295616.1	17	61733069	0.310	-	NAA25	NAA25
OAR17_67295616.1	17	61733069	0.310	-	TRAFD1	
s08464.1	24	35991517	0.307	-	MEPCE	
s08464.1	24	35991517	0.307	-	PILRA	
s08464.1	24	35991517	0.307	-	RAN	
s08464.1	24	35991517	0.307	-	TSC22D4	
s08464.1	24	35991517	0.307	-	ZCWPW1	
s24597.1	14	49053815	0.304	-	LTBP4	LTBP4
s24597.1	14	49053815	0.304	-	NUMBL	
s24597.1	14	49053815	0.304	-	SPTBN4	
OAR18_27752153.1	18	26754749	0.303	-	TRPM1	TRPM1
s50255.1	3	220327385	0.301	-	WNT7B	
s01490.1	3	180726856	0.299	-	CARD10	CARD10
s01490.1	3	180726856	0.299	-	MFNG	
OAR2_156184412.1	2	147210213	0.299	-	PSMD14	
OAR2_156184412.1	2	147210213	0.299	-	TBR1	
OAR3_112640501.1	3	105894007	0.289	-	ANAPC1	ANAPC1
s51044.1	3	7462549	0.288	-	CCBL1	

s51044.1	3	7462549	0.288	-	DOLK	
s51044.1	3	7462549	0.288	-	LRRC8A	LRRC8A
s51044.1	3	7462549	0.288	-	NUP188	
s51044.1	3	7462549	0.288	-	PHYHD1	
s49314.1	7	57002225	0.277	-	GABPB1	
s49314.1	7	57002225	0.277	-	HDC	HDC
s49314.1	7	57002225	0.277	-	SLC27A2	
OAR1_177805968.1	1	164847462	0.277	-	CCDC137	CCDC137
OAR5_22783606.1	5	20089099	0.277	-	ACSL6	ACSL6
OAR5_22783606.1	5	20089099	0.277	-	FNIP1	
s71728.1	17	71871881	0.274	-	HIRA	
s71728.1	17	71871881	0.274	-	MRPL40	MRPL40
s71728.1	17	71871881	0.274	-	UFD1L	
s28647.1	10	36317262	0.264	-	GJA3	
s28647.1	10	36317262	0.264	-	GJB2	
s28647.1	10	36317262	0.264	-	ZMYM2	ZMYM2
s25289.1	4	68802676	0.264	-	EVX1	
s25289.1	4	68802676	0.264	-	HOXA11	
OAR6_40370293.1	6	36155169	0.262	-	HERC3	HERC3
OAR6_40370293.1	6	36155169	0.262	-	HERC5	
OAR6_40370293.1	6	36155169	0.262	-	PREY	
s51433.1	17	12876687	0.261	-	ABCE1	
s51433.1	17	12876687	0.261	-	ANAPC10	
s51433.1	17	12876687	0.261	-	OTUD4	OTUD4
OAR21_45614480.1	21	41348152	0.260	-	ATL3	
OAR21_45614480.1	21	41348152	0.260	-	RTN3	RTN3
OAR7_63814443.1	7	57875478	0.258	-	FGF7	FGF7
OAR14_46482530.1	14	44143535	0.258	-	GPI	
OAR14_46482530.1	14	44143535	0.258	-	PDCD2L	
OAR14_46482530.1	14	44143535	0.258	-	UBA2	UBA2
OAR14_46482530.1	14	44143535	0.258	-	WTIP	
s10721.1	4	94145887	0.257	-	CPA1	
s10721.1	4	94145887	0.257	-	CPA4	
s10721.1	4	94145887	0.257	-	CPA5	CPA5
s10721.1	4	94145887	0.257	-	TSGA14	
OAR7_63960135.1	7	58027024	0.254	-	COPS2	
OAR7_63960135.1	7	58027024	0.254	-	GALK2	GALK2
s24740.1	2	110941391	0.253	-	PALLD	PALLD
OAR15_53192586.1	15	48728528	0.253	-	OR51E2	
OAR15_53192586.1	15	48728528	0.253	-	OR51G1	
OAR15_53192586.1	15	48728528	0.253	-	OR51I2	
OAR15_58554489.1	15	53412021	0.253	-	UVRAG	UVRAG
OAR3_200394051.1	3	186159798	0.252	-	CCDC91	CCDC91

Table S6. List of a priori function candidate genes

Bos taurus specific gene

ABAT	CAPN2	E1B976#	HBG2	MMP2	PLOD3#	TRH
ACE	CASP1	E1BIF9#	HBM	MT3	PML	TWIST1#
ACTN4	CASR#	E1BP33#	HBQ1	MTOR#	PPARA	TXN
ADA	CAV1	ECE1	HBZ	MYOD1#	PRKAA1	TXN2
ADAM17	CCL2	ECSOD#	HIF1A	NARFL	PRKCE#	TXNDC2
ADAM8#	CD34#	EDN1	HIF1AN#	NDRG1#	PRKCQ	UBE2B
ADIPOQ	CD38	EDNRA	HIF3A	NF1	PSEN2	UBQLN1
ADM	CDKN1A	EDNRB	HMOX1	NGB	PTEN	UCN
ADORA1	CFTR	EGFR	HMOX2	NKX3-1#	PTGIS#	UCN3
ADORA2A	CHRNA4	EGLN1	HRH1	NOS1	PTGS2#	UCP3
ADORA2B	CHRNA7	EGLN2	HSD11B2	NOS2	PTK2B	USF1
ADRB1	CHRNB2	EGLN3	HSP90AA1	NOS3	PTX3	VASN#
ADRB2	CITED2	ENG	HSP90AB1	NOTCH1#	PYGM	VEGFA
AGTR1	CLDN3	EP300	HSP90B1	NOX4	RORA	VHL
AJUBA#	CPS1	EPAS1	HYOU1	NPPB	RORB	VHLL
AKT1	CREBBP	EPHX2	ICAM1	NPPC	RORC	VLDLR
AKT2	CRYAA#	EPO	IFNG	NPR1	RYR1	XRCC1
AKT3	CRYAB#	ERCC3	IL10	NQO1	RYR2	
ALAS2#	CXCR4	F1MP20#	IL18	NR4A2	SCNN1B	
ALB	CYB5R4	F1MU57#	IL1B	OXTR	SCNN1G	
ALDH2	CYGB	F1N5F6#	INDO	P02081#	SERPINA1	
ALDOC	CYP17A1	F6R1H3#	INS	P2RX3	SFRP1#	
ALKBH5#	CYP19A1	FABP1#	INSR	P2RX4	SHH	
ANG	CYP1A1	FAM162A#	ITGA1	PDE5A	SIRT1#	
ANG1#	CYP1A2	FAS#	ITGA2	PDGFA	SLC11A2	
ANGPT1	CYP1B1	FLT1	ITPR1	PDGFB	SLC2A8	
ANGPTL4	CYP26A1	FRAP1	ITPR2	PDGFRA	SLC8A1	
APOE	CYP2A7	FUNDC1#	JAG2	PDIA2	SMAD3	
APOLD1	CYP2B6	G3MXR8#	JAK2	PDK1#	SMAD4	
AQP1#	CYP2C18	G3MYW0#	KCNA5	PDK3#	SMAD9	
ARG2	CYP2C19	G3MZ21#	KCNJ8	PDLIM1	SOCS3	
ARNT	CYP2C8	G3N1Y3#	KCNMA1	PDPN	SOD1	
ARNT2	CYP2C9	G3N3D8#	KLRK1	PGF	SOD2	
ASCL2	CYP2E1	G8JKV7#	KNG1	PIK3C2A	SOD3	
ATG5	CYP2F1	GATA6#	KNG2#	PIK3C2B	SPR	
ATP1B1	CYP2U1	GCH1	LCT	PIK3C2G	SRF#	
BACH1#	CYP3A4	GCHFR	LIMD1#	PIK3C3	STAT5B	
BAD#	CYP3A5	GIMAP1	LMNA#	PIK3CA	TDO2	
BBS2#	CYP3A7	GIMAP5	LOC100850173#	PIK3CB	TFRC	
BCL2	CYP4A11	GPR182	LOC784935#	PIK3CD	TGFB1	
BCL2L1	CYP4B1	GPX1	LONP1	PIK3CG	TGFB2	

BDKRB2#	CYP4F12	GUCY1A3	LOXL2#	PIK3R1	TGFB3
BIRC2	CYP4F2	HAAO	MB	PIK3R2	TGFBR1
BMP2#	CYP4F3	HBA#	MDM2#	PIK3R3	TH
BNIP3	CYP8B1	HBA1	MDM4#	PIP3-E	THBS1
CA9	DDAH1	HBB	MGARP#	PLAT	TICAM1
CABC1	DDAH2	HBD	MGC127538#	PLAU	TNF
CALCA	DDIT4	HBE1	MGC148992#	PLK3#	TP53#
CALCB	DPP4	HBE2#	MKKS#	PLOD1	TPTE
CAMK2D	DRD1#	HBE4#	MMP14	PLOD2	TPTE2

Table S7. Six hematologic parameters information for TIB and LTH breeds

Parameter	TIB(male) (n=16)	TIB(female) (n=14)	LTH(male) (n=16)	LTH(female) (n=15)
Red blood cell (RBC)(10^6/ul)	9.69 ±1.37 [a]	10.3 ±1.64 [a]	10.77 ±1.85 [a]	10.66 ±1.55 [a]
Hemoglobin (HGB)(g/L)	106.38 ±12.25 [a]	110.29 ±13.56 [a]	101.25 ±17.82 [a]	100.33 ±14.34 [a]
Hematocrit (HCT)(%)	33.93 ±3.31 [a]	34.57 ±3.47 [a]	32.12 ±5.47 [a]	31.51 ±4.75 [a]
Mean corpuscular volume (MCV)(fl)	35.28 ±2.63[a]	33.99 ±3.35[a]	29.88 ±1.48[b]	29.64 ±1.66[b]
Mean corpuscular hemoglobin (MCH)(pg)	11.02 ±0.62[a]	10.79 ±0.8[a]	9.43 ±0.47[b]	9.45 ±0.43[b]
Mean corpuscular hemoglobin concentration (MCHC)(g/L)	313 ±13.59 [a]	318.21 ±10.87 [a]	315.19 ±8.67 [a]	318.8 ±6.76 [a]

Note: The means with the same letter within the same row are not significantly different (P > 0.05)

Table S8. Summary of variation consequences in EPAS1 gene

ID	Chr:position(bp)	Exon	Alternative nucleotides		Amino acid(AA)	AA coordinate
No.1	77888694	13	T/C	AGT/AGC	S/P	423
No.2	77888602	13	A/G	CTA/CTG	L	453
No.3	77888574	13	A/G	AGC/GCC	A/T	463
No.4	77888547-77888533	13	INS[AGC]		SSSSS/SSSSSS	471-476
No.5	77886512	15	G/A	GAG/GAA	E	547
No.6	77886491	15	G/C	CCG/CCC	P	554
No.7	77886337	15	T/C	TTC/CTC	F/L	606
No.8	77886329	15	C/T	GAC/GAT	D	608
No.9	77884639	16	G/A	GAG/GAA	E	714
No.10	77884311	17	C/T	GAC/GAT	D	758
No.11	77881491	19	C/T			
No.12	77881485	19	G/A			

Table S9. The MAF and X^2 for each locus of TIB and LTH breed

ID	TIB					LTH					X-squared	p-value
	Genetype			Minor Allele Frequency (MAF)	H-W(P-value)	Genetype			Minor Allele Frequency (MAF)	H-W(P-value)		
No.1	29 (TT)	1 (TC)	- (CC)	0.02	0.01 (0.93)	13 (TT)	15 (TC)	3 (CC)	0.34	0.2 (0.66)	21.3346	0.0000
No.2	26 (AA)	3 (AG)	1 (GG)	0.08	3.58 (0.06)	11 (AA)	15 (AG)	5 (GG)	0.40	0 (0.98)	16.7359	0.0002
No.3	1 (AA)	2 (AG)	27 (GG)	0.07	6.47 (0.01)	- (AA)	4 (AG)	27 (GG)	0.06	0.15 (0.7)	1.6507	0.4381
No.4	26 (WW)	3 (WI)	1 (II)	0.08	3.58 (0.06)	27 (WW)	4 (WI)	- (II)	0.06	0.15 (0.7)	1.1456	0.5639
No.5	28 (GG)	2 (GA)	- (AA)	0.03	0.04 (0.85)	11 (GG)	15 (GA)	5 (AA)	0.40	0 (0.98)	22.341	0.0000
No.6	29 (GG)	1 (GC)	- (CC)	0.02	0.01 (0.93)	27 (GG)	4 (GC)	- (CC)	0.06	0.15 (0.7)	0.8017	0.3706
No.7	29 (TT)	1 (TC)	- (CC)	0.02	0.01 (0.93)	27 (TT)	4 (TC)	- (CC)	0.06	0.15 (0.7)	0.8017	0.3706
No.8	23 (CC)	5 (CT)	2 (TT)	0.15	3.6 (0.06)	19 (CC)	12 (CT)	- (TT)	0.19	1.79 (0.18)	5.2483	0.0725
No.9	- (GG)	5 (GA)	25 (AA)	0.08	0.25 (0.62)	- (GG)	- (GA)	31 (AA)	-	-	3.631	0.0567
No.10	23 (CC)	5 (CT)	2 (TT)	0.15	3.6 (0.06)	20 (CC)	11 (CT)	- (TT)	0.18	1.44 (0.23)	3.2041	0.2015
No.11	6 (CC)	16 (CT)	8 (TT)	0.47	0.15 (0.7)	18 (CC)	10 (CT)	3 (TT)	0.26	0.77 (0.38)	6.216	0.0447
No.12	12 (GG)	15 (GA)	3 (AA)	0.35	0.29 (0.59)	20 (GG)	10 (GA)	1 (AA)	0.19	0.03 (0.85)	3.9847	0.1364

Table S10. PCR primers used in this study

primer name	Sequence	Production length (bp)
EPAS1F1	gcacctggcacctttcac	289
EPAS1R1	atccctgggtcctcaaaatc	
EPAS1F2	ggcagtgatgttctggagact	300
EPAS1R2	tcctgcgtgtgagaactcg	
EPAS1F3	caccccctgtctttccag	697
EPAS1R3	ggaaagaaacacaccgaaaa	
EPAS1F4	ccactacccttctccatcca	392
EPAS1R4	accacaggcacaaccacag	
EPAS1F5	atctccccaccttgcttttt	213
EPAS1R5	ccttctcaccggtcacattt	
EPAS1F6	gcttcctctcgttgaccttg	1388
EPAS1R6	gagaaggtacacggcagctc	
EPAS1F7	cgtgttcttgagaagccaca	1446
EPAS1R7	ggtaaagcagaaggcagacg	
EPAS1F8	ccactgctgagctgctttg	587
EPAS1R8	agtgggcacctgtctgtacc	
EPAS1F9	ctctcacgcccactctctct	939
EPAS1R9	gctgagatctgccacctttc	
EPAS1F10	cacggttgattcagggtttt	566
EPAS1R10	aaggcgaacaaacgtacacc	

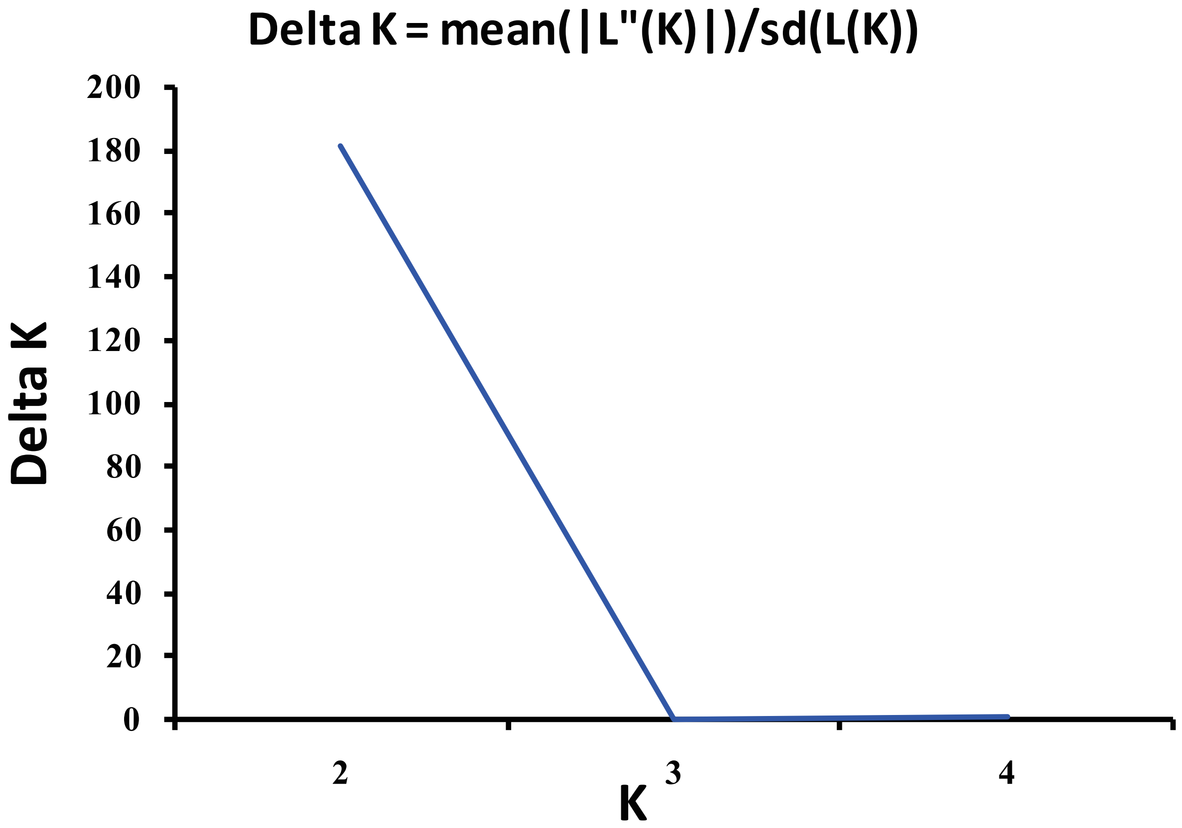

Figure S1. Mean L(K) (±SD) for each K value

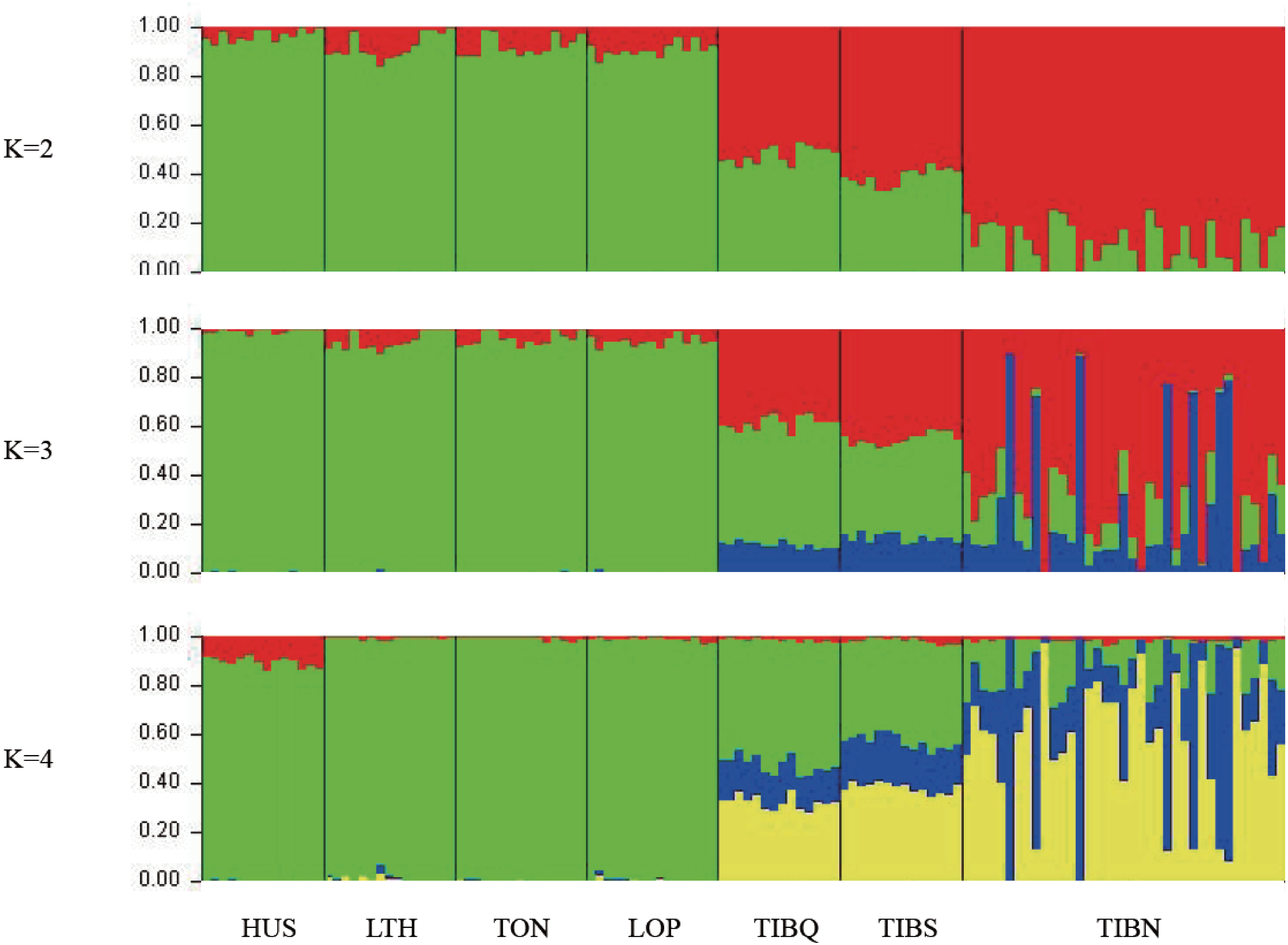

Figure S2. Structure analysis on all the individuals with K=2-4. The abbreviations for the 7 China native breeds are shown in Supplemental table S1
